# Evaluating methods for integrating single-cell data and genetics to understand inflammatory disease complexity

**DOI:** 10.3389/fimmu.2024.1454263

**Published:** 2024-12-05

**Authors:** Hope A. Townsend, Kaylee J. Rosenberger, Lauren A. Vanderlinden, Jun Inamo, Fan Zhang

**Affiliations:** ^1^ Biofrontiers Institute, University of Colorado Boulder, Boulder, CO, United States; ^2^ Department of Molecular, Cellular, Developmental Biology, University of Colorado Boulder, Boulder, CO, United States; ^3^ Department of Ecology and Evolutionary Biology, University of Colorado Boulder, Boulder, CO, United States; ^4^ Department of Medicine, Division of Rheumatology, University of Colorado Anschutz Medical Campus, Denver, CO, United States; ^5^ Department of Biomedical Informatics, Center for Health AI, University of Colorado Anschutz Medical Campus, Denver, CO, United States

**Keywords:** scRNA-seq, GWAS, SNP-gene linking, autoimmune diseases, benchmarking, omics

## Abstract

**Background:**

Understanding genetic underpinnings of immune-mediated inflammatory diseases is crucial to improve treatments. Single-cell RNA sequencing (scRNA-seq) identifies cell states expanded in disease, but often overlooks genetic causality due to cost and small genotyping cohorts. Conversely, large genome-wide association studies (GWAS) are commonly accessible.

**Methods:**

We present a 3-step robust benchmarking analysis of integrating GWAS and scRNA-seq to identify genetically relevant cell states and genes in inflammatory diseases. First, we applied and compared the results of three recent algorithms, based on pathways (scGWAS), single-cell disease scores (scDRS), or both (scPagwas), according to accuracy/sensitivity and interpretability. While previous studies focused on coarse cell types, we used disease-specific, fine-grained single-cell atlases (183,742 and 228,211 cells) and GWAS data (Ns of 97,173 and 45,975) for rheumatoid arthritis (RA) and ulcerative colitis (UC). Second, given the lack of scRNA-seq for many diseases with GWAS, we further tested the tools’ resolution limits by differentiating between similar diseases with only one fine-grained scRNA-seq atlas. Lastly, we provide a novel evaluation of noncoding SNP incorporation methods by testing which enabled the highest sensitivity/accuracy of known cell-state calls.

**Results:**

We first found that single-cell based tools scDRS and scPagwas called superior numbers of supported cell states that were overlooked by scGWAS. While scGWAS and scPagwas were advantageous for gene exploration, scDRS effectively accounted for batch effect and captured cellular heterogeneity of disease-relevance without single-cell genotyping. For noncoding SNP integration, we found a key trade-off between statistical power and confidence with positional (e.g. MAGMA) and non-positional approaches (e.g. chromatin-interaction, eQTL). Even when directly incorporating noncoding SNPs through 5’ scRNA-seq measures of regulatory elements, non disease-specific atlases gave misleading results by not containing disease-tissue specific transcriptomic patterns. Despite this criticality of tissue-specific scRNA-seq, we showed that scDRS enabled deconvolution of two similar diseases with a single fine-grained scRNA-seq atlas and separate GWAS. Indeed, we identified supported and novel genetic-phenotype linkages separating RA and ankylosing spondylitis, and UC and crohn’s disease. Overall, while noting evolving single-cell technologies, our study provides key findings for integrating expanding fine-grained scRNA-seq, GWAS, and noncoding SNP resources to unravel the complexities of inflammatory diseases.

## 1 Introduction

The efficacy of treatments for immune-mediated inflammatory diseases, such as rheumatoid arthritis (RA) and ulcerative colitis (UC), varies across patients (1). Single-cell RNA sequencing (scRNA-seq) technology enables the development of effective treatments for patients with immune-mediated inflammatory diseases by allowing the identification of specific cell states expanded in diseased tissue or blood (2). However, most scRNA-seq analyses do not consider genetic causality, and due to its high expense, available single cell datasets are often confined to small patient cohorts. Understanding the genetic underpinnings of diseases is key for preventative care, unraveling physiological and environmental contributions to pathology, and allowing personalized treatments. Genome wide association studies (GWAS) have been the gold standard to identify disease-associated genetic *loci* and summary statistics for large cohorts are often publicly accessible (3). Therefore, recent work has gone into combining the physiological insights from scRNA-seq with genetic associations from GWAS for unraveling disease causality (4–10). Indeed, attempts to integrate bulk RNA-seq studies with GWAS have been implemented, yet still only explain about 30% of the heritability by gene expression for complex traits (11). This pitfall is likely explained by the less fine-scale cell states available with bulk RNA-seq compared to scRNA-seq, where immune cells exhibit divergent expression profiles at nuanced cell states, and different cell phenotypes are uniquely associated with disease (12–14).

Recently, several computational tools have been developed to link disease relevant *loci* from GWAS to nuanced cell states revealed by scRNA-seq to identify disease-associated cell states and genes with both transcriptomic and genomic support (4–7, 9, 15). For each tool, major steps include summarizing variably expressed genes/pathways from single cell expression data, using a third-party method to link GWAS based single nucleotide polymorphisms (SNPs) to genes/pathways, and then using statistical tests to identify significant associations. However, a thorough comparison and assessment of these tools is lacking. Additionally, a critical step for all these tools, linking SNPs from GWAS to the genes they potentially impact, has been challenging with no clear solution (16–20). With more than 90% of immune-disease associated SNPs falling into noncoding regions, most of which are in cis-regulatory regions, the need to link these SNPs to physiological mechanisms cannot be overstated (21). The most common method for linking SNPs to genes does so according to a user-selected window size outside the gene. MAGMA, one of the most common tools that does this, can take both genotype data and summary statistics as input while accounting for Linkage Disequilibrium (16). It outputs a list of thousands of genes with the corresponding GWAS statistics reestablished at the gene level. However, many target genes of cis-regulatory regions are not the closest gene and can even be farther than 1 Mb away, contradicting the assumptions of tools like MAGMA (18). Therefore, alternative methods focusing on eQTL, chromatin contact (e.g. Hi-C), and similarly relevant enhancer-gene linking data have been introduced (17, 22). Additionally, newer studies have begun introducing single-cell transcriptomics methods that measure cis-regulatory elements to directly consider noncoding SNPs (10). The influence of incorporating noncoding SNPs using non-positional compared with positional methods, specifically within the context of these algorithms, has not been formally evaluated.

Beyond SNP-gene linking complexities, transcriptomics-genomics integration algorithms have currently been assessed for capturing broad associations (e.g. metabolic cells for metabolic diseases) (4, 5, 9). This limited analysis is primarily due to the usage of non-disease specific scRNA-seq atlases rather than disease-specific atlases with highly refined cell states identified. Disease specific, scRNA-seq atlases are quickly being developed and revolutionizing the understanding of diseased tissue heterogeneity. Yet the ability for tools tested on broader cell types to work with these more refined atlases with disease confounders has not been tested. Additionally, these tools might still be usable for diseases without atlases currently available by using atlases of similar diseases but the appropriate GWAS summary statistics.

Overall, despite the recent influx of tools integrating genetics and single-cell transcriptomics, a thorough comparison and assessment of different types of recent algorithms and major challenges of the domain is lacking. To address this, we conducted a benchmark analysis of the three most recent, open-source algorithms, scGWAS, scPagwas and scDRS, by objectively linking GWAS data with single-cell phenotypes across four immune-mediated disease datasets (4, 5, 9, 14, 23). We further annotated our results based on literature support of calls (detailed in Methods and **Supplementary Tables 1**, **2**), and evaluated the computational efficiency and result interpretability. Given most immune relevant SNPs are noncoding, we then evaluated the influence of different methods incorporating these SNPs for use in the algorithms (16, 17). As a result, we first showed that all three tools successfully identified expected significant cell types for tested diseases when using fine-grained scRNA-seq atlases, although with varying consistency and agreement. Single-cell scoring tools scDRS and scPagwas identified more significant results with literary support, although pathway-based scPagwas invokes a higher computational cost and cannot effectively consider batch effects. We also found that scDRS can be used to distinguish cell phenotypes for different diseases while using the same fine-grained scRNA-seq atlas. Finally, we provided evidence supporting the usage of positional based methods to incorporate noncoding SNPs until other methods can increase in statistical power and include more relevant atlases. Overall, our in-depth benchmarking and application on disease-tissue data demonstrated that current tools could identify associations between cell phenotypes and disease with high resolution and specificity. Our work pinpoints the capabilities and benefits of using atlases with fine-grained cell subtype annotations, while also showing that a single atlas could still be used to understand multiple diseases.

## 2 Materials and methods

**Figure 1:**
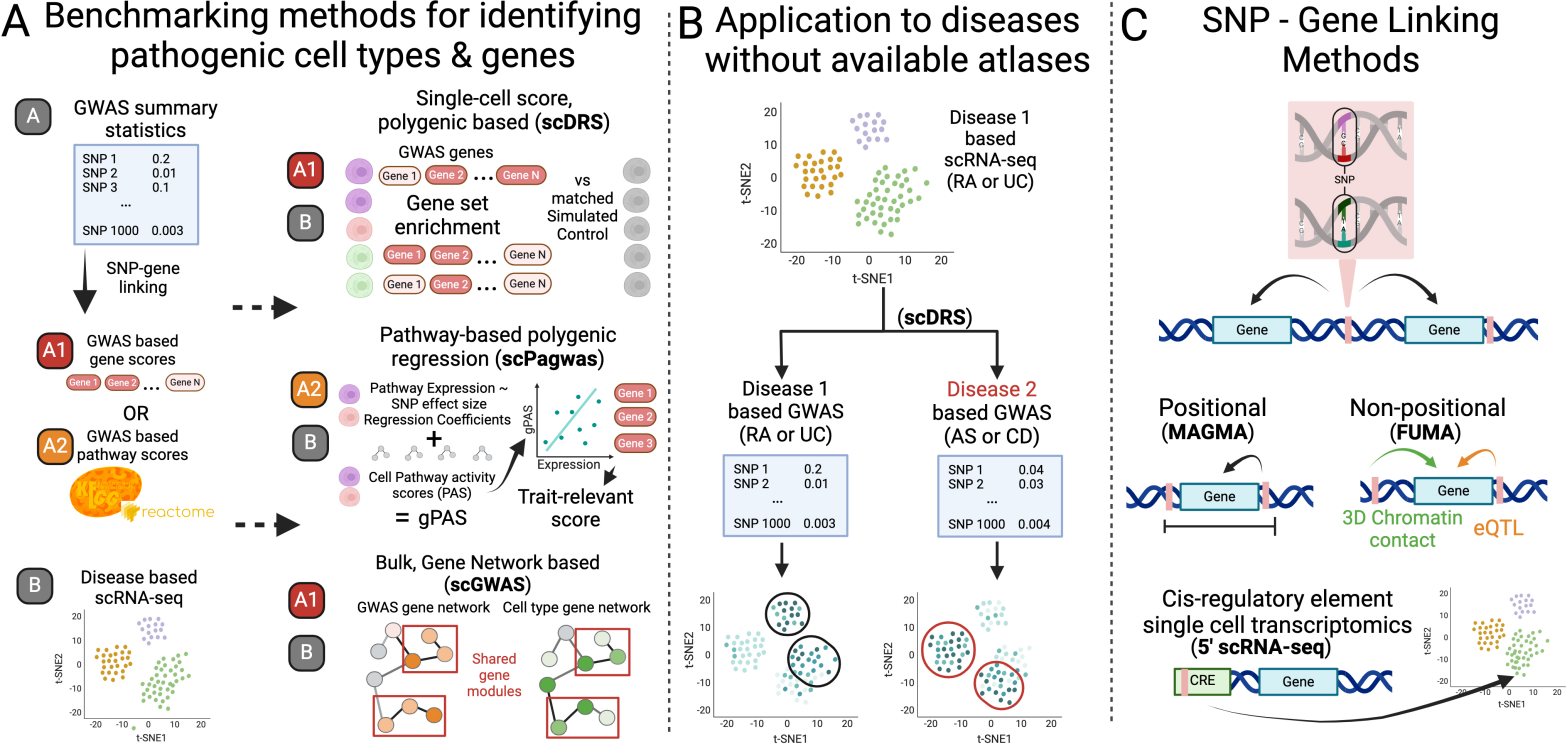
Overview of study design. **(A)** We first benchmarked the three most recent tools built to identify cell states and genes associated to disease according to both genetics (GWAS) and transcriptomics (scRNA-seq). **(B)** We next assessed if a single scRNA-seq atlas could be used with summary statistics from two diseases to reveal well separated disease associated cell states of the different diseases. **(C)** Finally, we assessed the robustness and accuracy of results of these tools when using different SNP-Gene linking methods. Figure made in Biorender.

We first benchmarked the three most recent and representative algorithms in the field according to the number of literature supported clusters called significant, computational efficiency, and result interpretability (**Figure 1A**). Brief descriptions of the tools can be found in sections 2.1 and 2.4. Expected results were based on a literature search for each individual cell phenotype for expansion in a disease and/or genetic connections, the results of which can be found in **Supplementary Tables 1** and **2**. If a general cell state with multiple, more detailed cell states was significant, the cell states were marked as having “general” literature support while if a specific cell state was supported, it had “specific” literature support. Due to the robustness of the available atlases and studies, we used scRNA-seq data generated from inflamed RA synovial and UC colon to determine disease-associated cell states (14, 23). Next, we assessed the feasibility of using identical scRNA-seq atlases to distinguish between two clinically similar diseases, using RA inflamed synovial tissue for RA and ankylosing spondylitis (AS), and UC colon for UC and Crohn’s disease (CD) (**Figure 1B**). Finally, we evaluated the incorporation of noncoding SNPs when using positional (MAGMA) vs non-positional based SNP-gene linking methods or cis-regulatory element focused single-cell omics like ATAC-seq or 5’-scRNA-seq (**Figure 1C**). We deploy all the code and analytical pipelines at our Github repository for reproducible research at https://github.com/fanzhanglab/SCRNA-GWAS-Benchmarking.

### 2.1 Selection of tools

We summarized the attributes of six currently available and supported packages that integrate scRNA-seq data and GWAS summary statistics to identify significant cell types and/or the GWAS-linked genes that best explain these cell types (**Table 1**). Other methods like RolyPoly, CocoNet, and sc-linker are described in **Supplementary Table 3**, and are either no longer maintained or not designed as user-friendly packages but instead open-source code (22, 24, 25). Briefly, RolyPoly was one of the first tools to employ the use of polygenic modeling to identify trait-relevant cell states, CocoNet pioneered gene-network based analyses, and sc-linker leveraged enhancer-gene linkages to assign SNPs to genes. The three tools chosen for more detailed benchmarking were the most recent tools and provide unique results as either gene-gene networks or single-cell based scores. The other methods differ most by their incorporation of noncoding SNPs which is addressed separately in this work.

**Table 1:** Summary table of the currently maintained and operable packages for identifying significant cell types and/or genes based on the integration of GWAS and single-cell RNA-seq data.

Package (Citation) Interface	Inputs	Relevant Outputs	SNP-Gene Linking	Summary	Highlights
scPagwas (9) R package	1. Seurat Object 2. GWAS summary stats	1. Cell score file 2. Cell Pathway Scores 3. Opt: Cell group score 4. Opt: Gene PCCs	Window-based	Pathway-based polygenic regression: linear regression of GWAS signals with pathway activation in cells.	Pathway-based while maintaining single-cell analysis
scGWAS (5) CL JAR, pre/post processing in R	1. Boxcox transformed gene p-values 2. Pseudobulk 3. Gene-gene network file	1. Significant gene modules in each cell type	Window-based: MAGMA	Network-based approach to identify cell types overexpressed with disease-significant genes	Pathway based for more meaningful output
scDRS (4) CLI or API	1. Anndata single cell expression data 2. Gene p-values or z-scores	1. Cell score file for a given trait 2. Opt: Cell group score and heterogeneity 3. Opt: Cell variable (e.g. gene) correlation to disease scores	Window-based: MAGMA	Monte Carlo simulation method that scores individual cells for disease association based on increased expression of sets of putative disease genes	Single-cell level allows unique post analyses
EPIC (6) R package	1. Pseudobulk gene expression 2. GWAS summary stats	1. Enrichment score of trait for each cell type 2. Relevant genes from DFBETAS	Sliding-window based LDSC	Gene-level chi-square association testing, then gene-level regression- association testing for each cell type	Adapted for rare and common variants
ECLIPSER (7) Scripts on Github	1. GWAS summary stats 2. Gene differential expression table	1. Prioritized cell types 2. Leading edge causal genes and eQTL impact	eQTL and other functional evidence	Cell-type specificity score for each GWAS locus, cell-type specific genes (from differential expression analysis mapped to locus)	Provides putative regulatory impact of genes
CELLECT (15) CLI	1. Specificity input from CELLEX 2. GWAS summary stats	1. Prioritized cell types 2. Opt: Gene heritability	LDSC or MAGMA	Heritability regression based method with CELLEX gene specificity scores	Allows easy usage of LDSC or MAGMA

A similar table for methods no longer maintained (RolyPoly) or not designed as packages for complete analysis workflows (CocoNet and SC-Linker) is available in **Supplementary Table 3**.

### 2.2 Data availability

The GWAS data used in this work can be found in **Supplementary Table 4**. Due to the most robust LD score data belonging to those with European descent, and the larger sample size of this group in both GWAS and scRNA-seq data, we focused on this subpopulation for the purpose of this benchmarking analysis. The major histocompatibility complex region was not included due to its complex genetic architecture. For GWAS summary statistics without rsids for RA, SNPs were assigned to rsids using BEDOPs and for duplicate/synonmous rsids, those with the lowest p-values were kept. The code for these steps can be found on our github under SCRNA-GWAS-Benchmarking/src/00B_Preprocess_GWAS.

For RA and AS, we analyzed a scRNA-seq data set developed by (14). To stay consistent with GWAS data, we only included cells from individuals of European descent with RA, leaving 183,742 cells. We used their most updated cell-state and cell-type annotations determined by their analysis of 314,011 cells with scRNA-seq, CITE-seq, experimental evidence and batch control to ensure the best validation. All expression was normalized with log(1 + UMIs for gene/totl UMIs in cell *10,000), and cells expressing fewer than 500 genes or that contained more than 20% if their total UMIs mapping to mitochondrial genes were removed. Further QC analysis is described in their paper (14). For UC and CD, we analyzed the scRNA-seq dataset from (23) which contained 228,211 cells passing quality control by using the raw counts and metadata they provide. For batch correction in both datasets, we applied Harmony, one of the best recommended methods for correcting for technical batch effect in single-cell batch data analysis and integration (26, 27). We used identical batch variables for correction as used in the original analysis for RA: the individual from which the cells were isolated (“sample”) (28). Combat was used for batch correction originally in Smillie et al., but is not designed for single-cell data, therefore we applied Harmony with “sample” to the UC scRNA-seq data instead (23, 29). Both scRNA-seq data only contained individuals of non-Hispanic, European descent. For scPagwas, we created Seurat objects with the same QC-based cells but using the Seurat based normalization. Due to the high computational expense of scPagwas, we excluded certain cell states from the RA and UC datasets that were not found significant by the literature on RA and UC, including Endothelial (RA & UC), Glia, Macrophages, TA 1 & TA 2(UC), and fibroblast cell states except for F-7: NOTCH3+ sublining and F-2: CD34+ sublining (RA). The code for these steps can be found on our github repository under SCRNA-GWAS-Benchmarking/src/00A_Preprocess_scRNA.

### 2.3 SNP-gene linking

MAGMA-based SNP-gene linking was done using version v1.10 with NCBI37.3.gene.loc and NCBI38.gene.loc downloaded from the MAGMA website as the gene locations files, and European UK Biobank Phase 3 LD scores. The window sizes of 10-10kb and 50-35kb were chosen for final comparison of significant cell states as the most common window size and that used in the original scGWAS paper, respectively. When assessing the impact of this window size parameter on scDRS, sizes 0kb, 5kb, and 100kb were also chosen based on the window sizes used across the literature (**Supplementary Table 5**). For this parameter stability assessment, the top-ranking genes according to MAGMA that were also found in the scRNA-seq expression data were used, with a final total of 1000 genes. Synonyms according to genecards.org and humanproteinatlas.com were also considered to verify proper comparison of genes between MAGMA and scRNA-seq. Genes from the scRNA-seq dataset still not found in the MAGMA file were added to allow their inclusion in the analysis. The genes identified by MAGMA but not found in scRNA-seq data are discussed further in the **Supplementary Material**, with numbers dictated in **Supplementary Table 6**.

The code for all these steps can be found on our github under SCRNA-GWAS-Benchmarking/src/01_MAGMA_Gene_Alias.

FUMA is a web-based tool that determines statistically significant disease associated genes using positional, eQTL, and 3D chromatin based mapping, but does not calculate a summary p-value like MAGMA (17). Therefore, to explore the implications of including these forms of mapping, we used the minimum GWAS SNP P-value (minGwasP in genes.txt output file) for each gene as a proxy for a disease-association p-value to allow input for scDRS and scGWAS. FUMA identifies lead SNPs, maps to rsIDs, addresses duplicate and synonymous rsIDs, and filters out the MHC region in its analysis from the summary statistics. Default parameters were used including a MAGMA window of 10kb, with MAGMA expression data being based on GTEx v8. We also used eQTL and Chromatin Interaction Mapping, both including the options of available blood cell eQTL data. Versions include FUMA v1.5.3, MAGMA v1.08, GWAScatalog e0_r2022-11-29, and ANNOVAR 2017-07-17.

### 2.4 scGWAS, scDRS, & scPagwas

scGWAS uses a network-based approach to uncover cell types that significantly express disease-associated genes and identify gene modules representing disease-specific processes (5). Unlike other methods where cell types are assigned a disease-significance score, scGWAS assigns significance scores to gene modules with strong representation in both scRNA-seq cell type expression and GWAS based on a proportional test (**Figure 1A**). scGWAS is implemented in Java via a JAR package (ver. scGWAS_r1.jar) on the authors’ GitHub repository (https://github.com/ElkonLab/scGWAS) and can be run through the command line. Based on author recommendations on their GitHub repository, configuration file parameters were kept at default values. Further, we first used the same PathwayCommons input network file as Jia et al. (5), with gene-gene relationship information used for constructing the background network. We also created a second PathwayCommons input network file following their same steps but with v14 rather than v12 (what they used originally). Briefly, housekeeping and ribosomal genes were removed as well as any genes within 50kb of one another (detailed jupyter notebook and output pathway file found on our github under SCRNA-GWAS-Benchmarking/data/Pathway). We followed the analysis pipeline described on the authors’ GitHub repository for the following steps. For the screen expression input file, we processed the scRNA-seq dataset using their R-script to calculate the average log-transformed gene-based CPM per defined cell type. We processed the MAGMA output using the box-cox transformation script as the GWAS node input file. We ran scGWAS on the same scRNA-seq dataset first with general cell types and then on fine-scale defined cell states. The code for these steps can be found on our github under SCRNA-GWAS-Benchmarking/src/03_scGWAS.

scDRS assesses disease-associations at the individual cell level using a gene set enrichment analysis with genes with scored associations to the trait of interest according to a third party method (4) (**Figure 1A**). It then presents downstream analyses that use unified Monte Carlo tests to identify significant pre-annotated cell states according to a group Z score, and the genes whose expressions correlate with disease scores. It is the only tool designed to take cell-level covariates to address potential batch effects. The CLI version (Version v102 v1.0.2) of scDRS was used according to their GitHub repository (https://github.com/martinjzhang/scDRS). All default parameter values were used, and P-value files output from MAGMA served as input to scdrs munge-gs. The covariates files used in computing scDRS scores included nUMI, number of genes, and sex for both RA & UC, and age and duration for RA, and sample location, percent of mitochondrial reads, and smoking status for UC (found in our github at SCRNA-GWAS-Benchmarking/data/SC_data). We ran downstream analyses to identify significant cell groups on the same scRNA-seq dataset using annotations of general cell types and then with fine-scale defined clusters. The code for these steps can be found on our github repository under SCRNA-GWAS-Benchmarking/src/02_scDRS.

scPagwas associates cells and cell types to traits through pathways rather than only individual genes, while maintaining associations at the individual cell level (9). Rather than using a pre-determined GWAS based gene set list with scores like scDRS and scGWAS, scPagwas calculates genetically associated pathway activity scores (gPAS). Briefly, the gPAS is the product of a per-cell coefficient of a linear regression between SNP effect sizes and gene expression within a pathway, and the pathway activity score of the cell (first principal component of an SVD). Finally, following a similar logic of scDRS, a trait-relevance score is calculated using the Seurat cell scoring method which considers the expression of the top 1,000 genes most correlated with the summed gPAS in cells (**Figure 1A**). We followed installation instructions from the scPagwas github (https://github.com/sulab-wmu/scPagwas) for version 1.3.1, using Seurat version 5.1.0 and SeuratObject version 5.0.2. Code for these steps can be found on our github repository under SCRNA-GWAS-Benchmarking/src/04_scPagwas. To run scDRS with scPagwas genes, the 1,000 genes with the highest Pearson correlation coefficient (PCC) values output by scPagwas were used without weights (scDRS automatically assumes all weights are 1 if none are provided) (4). The use of PCC values as weights did not lead to a significant difference, so only unweighted based results are discussed. Code to generate the scDRS input can be found in SCRNA-GWAS-Benchmarking/analysis/0A_Tool_Benchmarking/Genes/Gene_comparison.ipynb.

### 2.5 Benchmarking methods

All packages provide results indicating which cell clusters are significant for the disease, but the exact format and calculation of these results differs. scGWAS provides significance in the form of gene modules within clusters that have disease-relevance, whereas scDRS and scPagwas provide disease scores at the single cell and cluster levels. scDRS additionally provides measurements regarding the heterogeneity of these disease scores within each cluster. To compare results across the three packages, we defined significant cell clusters in scGWAS as clusters with at least one disease-significant gene module. We then assessed whether the packages identified significant cell types similarly across a given disease. We also evaluated possible bias of scores from the health status of individuals and the sensitivity of scDRS to different numbers of top-ranking MAGMA genes (100, 300, 500, 1000, 1500, 2000). Additionally, we assessed the change in results of scGWAS to different pathway files (details in scGWAS and scDRS section above) according to both the significant gene modules and significant cell-states. Jupyter notebooks outlining these comparisons can be found at our github under SCRNA-GWAS-Benchmarking/analysis/0A_Tool_Benchmarking/Sensitivity and CT_Clusters. We also compared the genes considered most linked to the traits by the tools: scGWAS gives the significant gene modules, scDRS gives the correlation of gene expression to disease scores, and scPagwas gives the PCCs of gene expression according to a singular value decomposition method to calculate pathway activity scores in cells. We assessed the expression and correlation of significant gene modules identified by scGWAS or MAGMA top-ranking genes with scDRS and scPagwas disease scores, and compared scDRS and scPagwas correlation coefficients under SCRNA-GWAS-Benchmarking/analysis/0A_Tool_Benchmarking/Genes. Finally, the relationship of scDRS heterogeneity scores with cell-state population sizes and granularity was done with code under SCRNA-GWAS-Benchmarking/analysis/0A_Tool_Benchmarking/.

To compare genes, we analyzed the top 1,000, 500, and 100 genes ranked by MAGMA, scDRS, and scPagwas, as well as all significant gene modules identified by scGWAS. Using Gene Set Enrichment Analysis (https://www.gsea-msigdb.org/gsea/msigdb/human/compute_overlap), we examined gene sets enriched across our genes belonging to the Cell type (C8) collection, just Curated Pathways (C2-CP), or a combination of Hallmark, Curated (C2), Regulatory (C3), Biological Process (GOBP), and IMMUNESIGDB (C7-IMMUNE) (30, 31). GSEA allows a maximum of 500 genes. We ran scGWAS with all significant gene modules collectively or individually for C8 to ensure logical results given the smaller gene numbers. We also conducted GO analysis with clusterProfiler_4.12.2 and org.Hs.eg.db_3.19.1 (32, 33). Code for this analysis can be found in SCRNA-GWAS-Benchmarking/analysis/0A_Tool_Benchmarking/Genes/Gene_comparison.ipynb.

To determine whether a single atlas could distinguish between two similar diseases, we ran scDRS on the RA and UC cell atlases using MAGMA results from summary statistics of AS and CD GWAS, respectively. The code for analyzing scDRS results for this can be found under SCRNA-GWAS-Benchmarking/analysis/0B_Dist_path. The code for analyzing the effects of using different MAGMA window sizes and FUMA can be found under https://SCRNA-GWAS-Benchmarking/analysis/0C_Preproc.

## 3 Results

### 3.1 Single-cell disease scores allow greater sensitivity while gene-network analyses allow greater interpretability of gene targets

We built our initial benchmarking pipeline on evaluating both cell types and finer grained cell states as well as gene modules using RA and UC datasets.

#### 3.1.1 Comparison of disease-significant cell types/cell states

**Figure 2:**
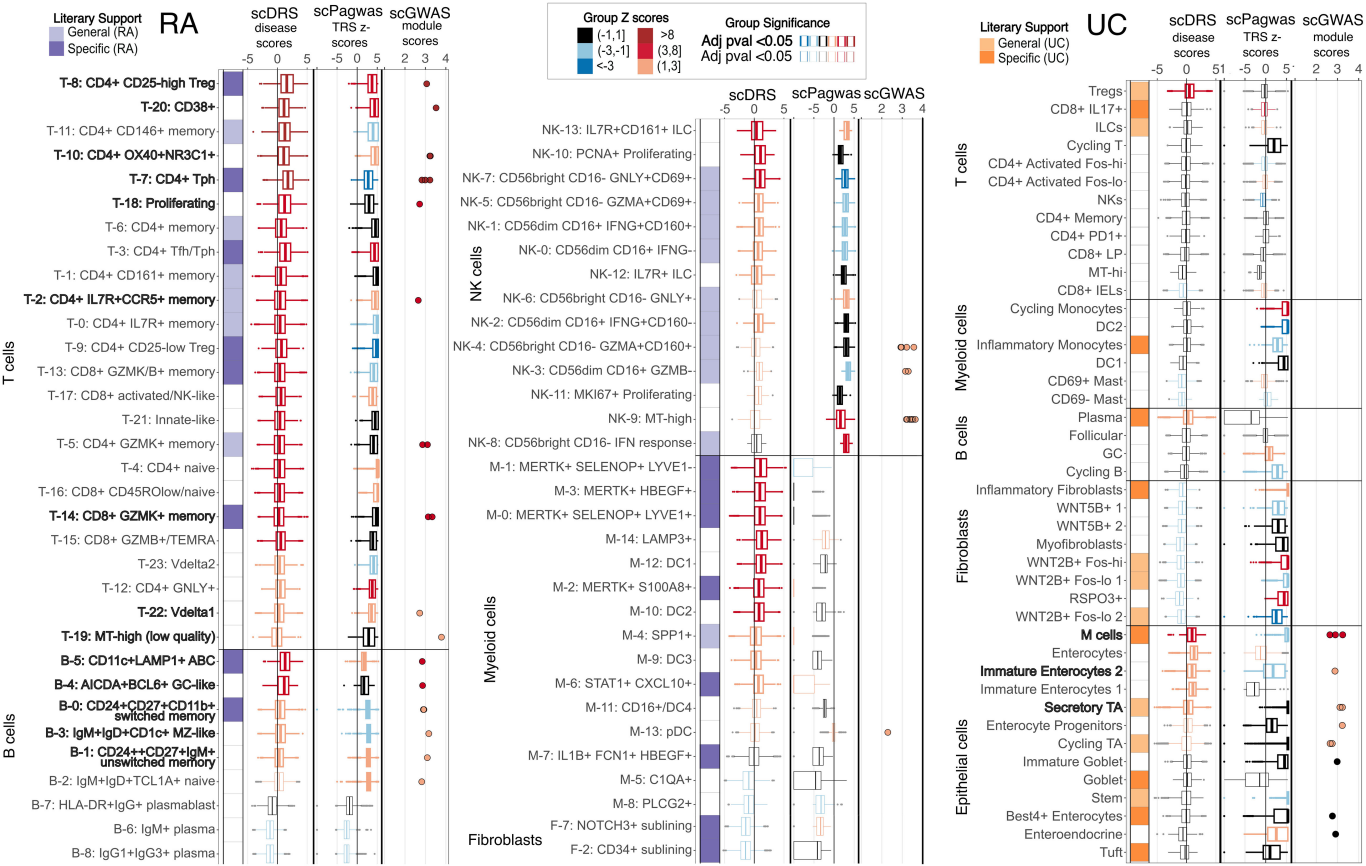
Comparison of cell-state-specific significance results for RA and UC. For each cell-type and cell-state, the single-cell level scDRS Z-scores and scPagwas TRS Z-scores are displayed in boxplots colored according to the group scDRS Z-score or group scPagwas bootstrap Z-score. Non-significant cell states in scDRS or scPagwas are shown unbolded with grey outliers, while significant cell states are bolded. scGWAS called gene modules and their disease scores are also plotted with colors following the scDRS group Z-score gradient for easier comparison. Cell states considered significant by all three tools are bolded. “General literary support” means the general cell type has been shown to associate with the disease while “specific” denotes evidence in the literature linking the specific cell state. Left: RA (rheumatoid arthritis). Right: UC (ulcerative colitis).

At the scale of cell types, all tools imply significance of NK cells in RA (**Supplementary Figure 1**). Both scDRS and scPagwas identified T cells as significant, while scPagwas and scGWAS identified B cells as significant. scDRS alone determined Myeloid cells to be significant for RA (**Supplementary Figure 1**). For more specific cell-states, the three tools shared the same significance calls for 24/63 (38%) fine-grained cell states. In general, all three tools identified significant cell states within the T and B cell compartments. This overlap was particularly notable in the results from scDRS and scPagwas. scGWAS called only 20 significant cell states (45% with literary support) compared to the 46 (54% with literary support) and 43 (53% with literary support) calls from scDRS and scPagwas (**Figure 2**). scDRS alone identified MERTK+ myeloid cell states as significant (14, 34, 35). scDRS still identified MERTK+ myeloid cell states as significant when using the same genes used by scPagwas (top 1000 correlated with gPAS cell scores) as input rather than the top 1000 MAGMA genes (**Supplementary Figure 2**). Additionally, scPagwas called all NK cell cell states significant for RA, while opposing subsets of NK cell states were called by scGWAS and scDRS (**Figure 2**).

There were a smaller number of significant cell types/states identified for UC. All tools identified epithelial cells as significant and T cells as not; all other cell types had mixed calls from tools (**Supplementary Figure 1**). For fine-grained cell states, all tools shared the same significance calls for 20/43 (47%), including M epithelial cells, Immature Enterocytes, and Secretory TA cells. Again, scPagwas called a high number of significant cell states (25, 44% with literary support) and was the only tool to identify most myeloid and fibroblast cell states as significant, including the inflammatory subtypes. scDRS and scGWAS showed similar numbers for significant cell states with seven (57% with literary support) and eight (50% with literary support), respectively (**Figure 2**). When running scDRS with the genes used by scPagwas, scDRS also identified the fibroblasts and non-mast myeloid cell states as significant (**Supplementary Figure 2**).

#### 3.1.2 Significant genes

**Figure 3:**
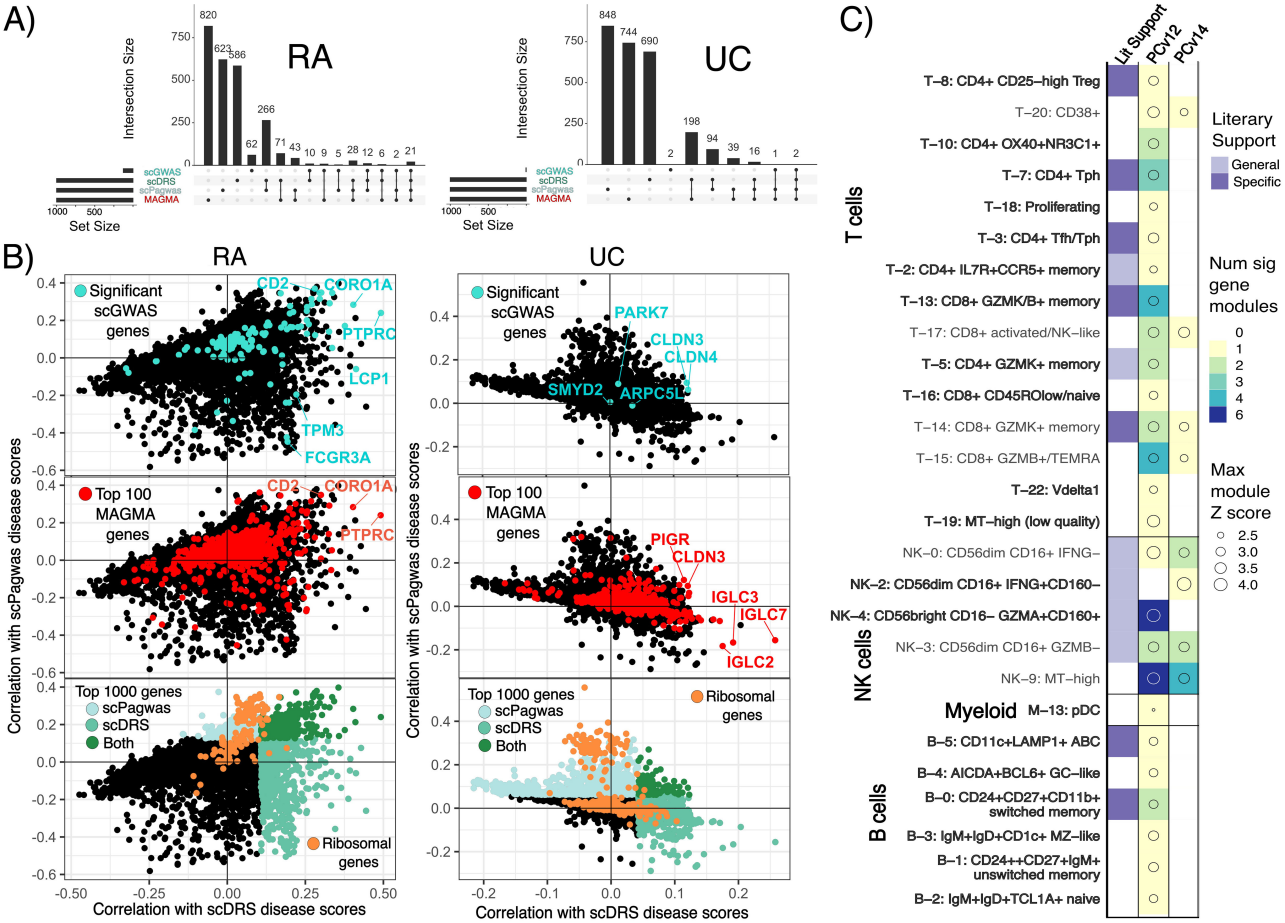
Gene comparisons show low correlation across tool-based genes and single-cell disease scores. **(A)** UpSet plots of the top 1000 ranked genes for scDRS (highest correlation to scDRS disease scores), scPagwas (highest correlation to genetically associated pathway activity scores) and MAGMA as well as the significant scGWAS genes. RA=Rheumatoid arthritis, UC=Ulcerative colitis. **(B)** Scatter plots of the correlations of all studied genes with scDRS disease scores and scPagwas gPAS with (top) scGWAS genes, (middle) MAGMA genes, or (bottom) ribosomal genes highlighted. Genes reaching the top 1000 ranked genes for scPagwas and scDRS are colored in light and dark turquoise, respectively. **(C)** scGWAS results when using a pathway file based on Pathway Commons v12 or 14. Results are highlighted according to the number of significant gene modules called per RA cell state and max disease Z score across the modules for each cell state. Only cell states with a significant gene module from using either pathway file are shown. Cell states without a significant gene module called when only one of the pathway files was used are bolded.

Significant modules identified by scGWAS are networks of genes that may represent a biological pathway and contain genes important for disease pathogenesis. scGWAS assesses these gene modules with each annotated cell type cluster. Notably, significant gene modules strongly align with functional annotations of their corresponding cell-states, as confirmed by gene set overlap analysis (30, 31) (**Supplementary Table 7**). For example, T cell gene modules were frequently enriched with cytotoxic or T helper cell surface molecules while gene modules associated with NK cell states were enriched in genes involved in upregulating CD4 T cells and cellular responses to cytokines, chemokines, and cellular ligands. Many of these gene modules had overlapping genes and similar functions; despite having a total of 204 and 472 genes in NK and T cell cluster significant modules, there were only 63 and 87 unique genes, respectively. One gene in particular was found in nearly every significant gene module across cell states–CD2, which encodes for a surface antigen in all T cells and is involved with triggering T cells (36). Both scDRS and scPagwas provide genes whose expressions correlate with the scDRS cell disease scores and scPagwas gPAS, respectively (4, 9). The majority (59-85%) of the top 1,000 scoring genes in MAGMA, scPagwas, and scDRS are unique to each tool, while 75-90% scGWAS significant genes are identified by at least one other tool (**Figure 3A**). Additionally, significant genes from MAGMA and scGWAS show low median correlations to scDRS and scPagwas disease Z-scores (MAGMA: 0.02,0.05 for RA and 0.02,0.01 for UC; scGWAS: 0.06,0.09 for RA and 0.04,0.01 for UC) (**Figure 3B**). For RA, scDRS, scGWAS, and MAGMA but not scPagwas top ranked genes were enriched in myeloid cell type genesets (**Supplementary Table 8**). For UC, all tools except scGWAS showed myeloid cell-specific gene set enrichment, with scPagwas being the only tool to show significant enrichment for stromal terms in the top 50 pathways (**Supplementary Table 9**). The top 100 ranking genes for scPagwas were largely ribosomal genes regardless of the disease (43% and 68% in RA and UC, respectively) while scDRS’s top 1,000 genes contained very few if any (**Figure 3B**). Indeed, the top 20 enriched gene ontology terms for scPagwas were related to translation or general differentiation while scDRS was dominated by leukocyte-specific pathways (**Supplementary Figure 3**). Gene sets uniquely enriched in scPagwas genes focused on translation, ribosomes, and general cell differentiation, unlike those specific to scDRS, MAGMA, and scGWAS which were immune-cell state or process focused (**Supplementary Tables 8**, **9**). Removal of the ribosomal genes when using scPagwas genes as input to scDRS only led to one and four cell states to change in significance in RA and UC, respectively, compared to scDRS results using all scPagwas genes (**Supplementary Figure 2**).

#### 3.1.3 Investigating result differences between pathway-based tools and scDRS

We first explored if variance in significant genes between methods might explain the different significant cell states identified by scGWAS and scDRS. We evaluated if the genes that most highly correlated with scDRS disease scores for cells in the MERTK+ cell states were found in networks in the original scGWAS pathway file and KEGG pathways. Indeed, pairs of genes that are strongly associated with scDRS disease scores were connected in the scGWAS pathway file, however, relationships between the genes beyond two were not supported and the 40 genes with the highest correlation to scDRS disease scores had only 6 pairings between them in the pathways file (**Supplementary Table 10**). The top 20 KEGG pathways uniquely enriched for MERTK+ cells according to scPagwas genetically associated pathway activity scores included Wnt signaling, cGMP-PKG signaling, and Inositol phosphate metabolism. We also explored the large discrepancy between NK calls across scGWAS and scDRS. As a controlled comparison, we looked at a cell cluster with strong agreement between scGWAS and scDRS: CD4+ Tph (T-7). scDRS disease scores in all cells positively correlated with the expression of the NK scGWAS module genes although T-7 scGWAS module genes had a slightly higher median correlation (0.08 vs 0.13) (**Supplementary Figures 4A, B**). This relative increase was maintained when the eight genes identified by scGWAS as significant for both groups were removed (median correlations 0.005 NK vs 0.02 T-7). Importantly, these correlations were comparable to that observed for all scGWAS genes and the top 100 genes ranked by MAGMA with scDRS disease scores (Medians of 0.01-0.09) (**Figure 3B**). Median correlations decreased when only considering cells within the corresponding cell states (NK-cells & T-7) unlike those of the top ranking scDRS genes for each cell state (**Supplementary Figures 4C, D**, **5**). These findings led us to assess the impact of the pathway file used by scGWAS on results. When using gene pairings from Pathway Commons v14 instead of v12 (see Methods for details), 20 RA and 8 UC cell states changed in whether they had at least one significant gene module identified. Of these, 13 RA cell states and 1 UC cell state had been originally called significant by scDRS, scGWAS, and scPagwas (**Figures 2**, **3C**, **Supplementary Figure 6A**). Extending the gene-SNP linking window from 10-10kb to 50-35kb resulted in 14 cell states no longer having a significant gene module (**Supplementary Figure 6B**). Despite having 319,042 more gene pairings, use of Pathway Commons v14 led to an overall decrease in significant gene modules called regardless of window size used. Even when cell states were called with both pathway files, the genes within significant gene modules were also dependent on pathway input despite all changing genes being found within both pathway input files (**Supplementary Figures 7**, **8**).

While scDRS single cell disease scores followed an expected normal distribution, disease scores from scPagwas or from scDRS run with scPagwas genes showed large polarization (**Figure 2**, **Supplementary Figures 2**, **9**). Specifically, 23% and 12% of cells in RA and UC, respectively, had scPagwas Z-scores of -10 despite the next nearest Z-score being -5. These percentages decreased to 17.5% and 3% when applying the scDRS framework to scPagwas genes, and further to 15% and 3% when ribosomal genes were removed for RA and UC, respectively. These cells were distributed across cell states, although most were found in plasma and MERTK+ cells for RA (**Supplementary Figures 9**, **10**).

Finally, although all tools may be impacted by covariates within the data, only scDRS allows for their inclusion for batch-effect analysis. In both RA and UC datasets, certain cell states contain significantly different proportions of cells from individuals according to health status (**Supplementary Figure 11**). scPagwas shows clear, significant differences in its single cell trait relevant scores, whereas scDRS exhibits minimal to no batch effects (**Supplementary Figures 12**, **13**). When scPagwas genes are used, biases in scDRS disease scores related to health status become more pronounced but remain less substantial than those in scPagwas disease scores (**Supplementary Figures 12**, **13**).

#### 3.1.4 Additional features

Although all scDRS additional features are outside the scope of this work, we evaluated the usage of the tools’ group-level metric to consider the heterogeneity of disease scores within a cell state (4). This metric can hypothetically indicate if a provided cell state has inner-clusters of cells that should be further separated out based on the groupings of disease score. All large-scale cell types in RA (T cell, B cell, Myeloid, NK, Fibroblast, Endothelial) had significant heterogeneous disease scores that positively correlated with the number of cells (adjusted R^2^ 0.29) and annotated clusters in each group (adjusted R^2^ 0.37) (**Supplementary Figure 14**). Eighty-seven percent (67/77) of RA fine-scale cell states had significant levels of heterogeneity in disease score with similarly low positive correlation with the number of cells (**Figure 2**, **Supplementary Figures 15**, **16**).

#### 3.1.5 Resources

Despite these additional features and working at the single-cell level, scDRS was the most robust in memory usage and speed, although this is primarily due to the initial preprocessing step for scGWAS (**Table 2**). scPagwas took the longest by 45 hours compared to scDRS and 32 hours compared to scGWAS (**Table 2**). Notably, the number and size of cell states had a negligible effect on resource usage in scDRS and scGWAS unlike scPagwas.

**Table 2:** Resource usage of each package when running for the RA cluster-level data.

Package	CPU used (time)	Wall clock time	Memory Used	Relevant Function (script)
scDRS	00:00:05	00:00:07	488 KB	Preprocess GWAS stats (run_scdrs.sh)
scDRS	00:54:13	00:38:43	12.26 GB	Compute single cell scores (run_scdrs.sh)
scDRS	00:23:41	00:25:11	17.89 GB	Cell-type scores & Gene analysis (run_scdrs.sh)
scGWAS	04:32:03	04:33:37	208.4 GB	Preprocessing single cell data (process_sc_data_R.sh)
scGWAS	08:50:26	08:50:24	2.55 GB	Running scGWAS (run_scGWAS_2023_clusters.sh)
scPagwas	1-16:48:25	1-21:47:25	185 GB	Running scPagwas
scPagwas		1-19:00:00		Link GWAS and Pathway block annotations

Memory used refers to the max amount of memory required for a single step. All tools were run with 15 CPUs.

### 3.2 scDRS can distinguish similar diseases from pathological cell clusters

While atlases with fine-grained annotations may allow more detailed analyses, it raises the question of whether a single atlas can still be used to study multiple diseases. This is particularly relevant for diseases without single-cell data available. Given the high sensitivity of single-cell disease scores, we used scDRS to assess the feasibility of using one atlas to identify pathological cell clusters distinguishing similar diseases. We used summary statistics from GWAS for RA and ankylosing spondylitis (AS) on the scRNA-seq data from inflamed RA synovial tissue to determine if scRNA-seq from a clinically similar disease can provide fine-grained insight on disease-relevant clusters (14, 37, 38). We also applied the GWAS statistics from UC and crohn’s disease (CD) on the scRNA-seq data from UC colon tissue (23, 39). We considered both 10-10kb and 50-35kb window sizes on these analyses, focusing main figures on 50-35kb window results due to the larger number of significance calls.

#### 3.2.1 RA and AS

**Figure 4:**
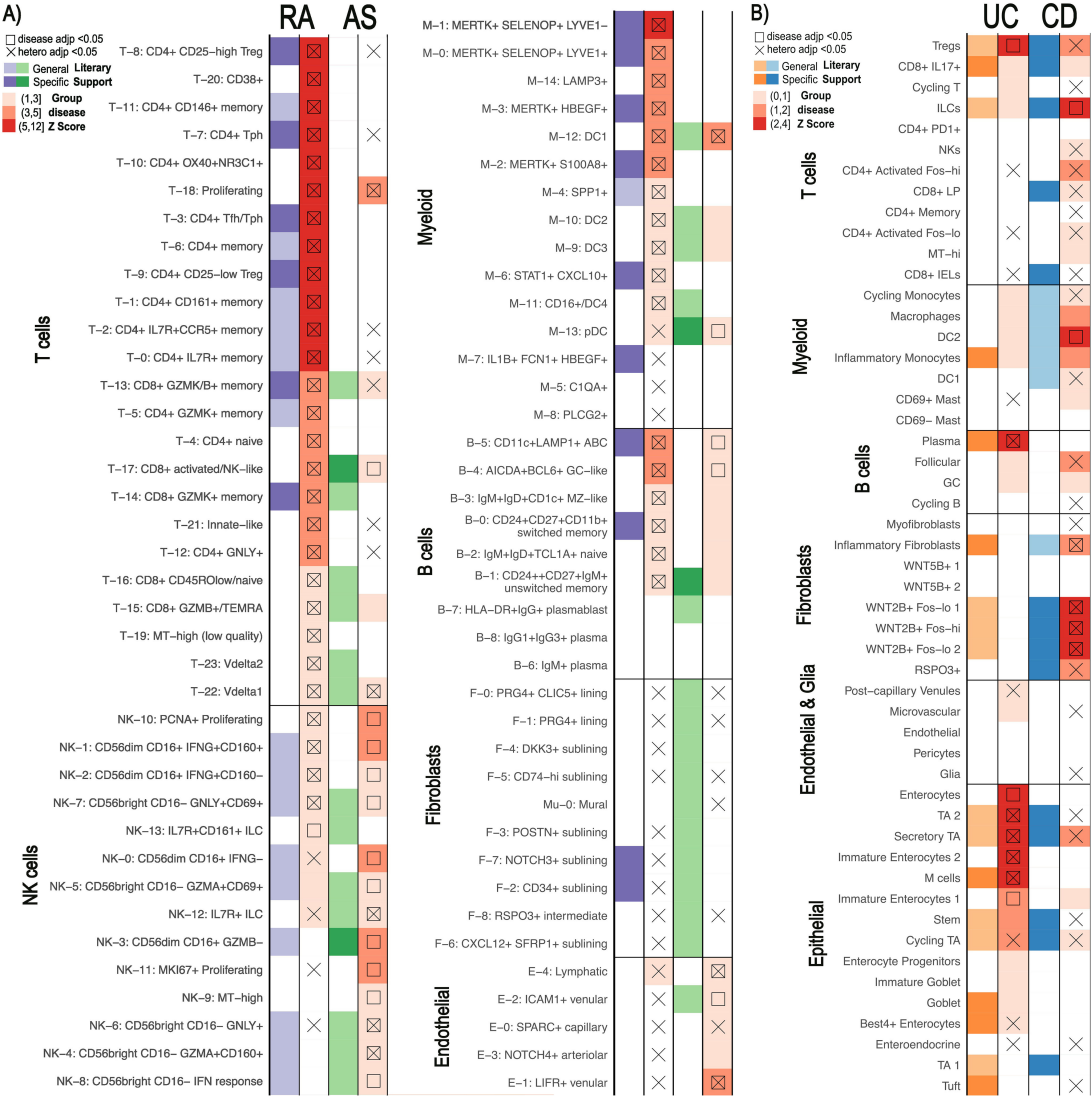
Comparison of similar diseases with scDRS. Summary statistics unique to each disease were used on the same scRNA-seq data for each pair (14, 23). scDRS defines significant clusters (annotated according to original papers) with a group disease Z-score as shown in the gradient legend. Cell clusters with literary support for either disease are labeled in purple/orange for RA/UC and green/blue for AS/CD, respectively. General literary support means that a cell type with multiple cell states is supported by the literature while specific means a specific single cell state was supported. **(A)** Rheumatoid arthritis (RA) vs Ankylosing Spondylitis (AS). **(B)** Ulcerative Colitis (UC) vs Crohn’s Disease (CD).

Although both analyses used the same scRNA-seq atlas references, scDRS successfully distinguished RA from AS. We identified 46 candidate cell clusters in RA and 23 in AS, with 10 clusters shared between the two diseases. We found that while most T, myeloid, and B cell cell-states were significant for RA, very few were significantly associated with AS (**Figure 4A**). CD8+ activated/NK-like (T-17), pDC (M-13), and unswitched memory cells (B-1) were significant for AS. AS and RA showed the greatest differences across the T, NK, and myeloid cells. While essentially all T cell states showed significance for RA, only CD8+ activated NK-like (T-17) and proliferating (T-18) T-cells showed significance for AS. Conversely, far more NK cell clusters were called significant for AS (43, 44). Specifically, most of the CD56bright CD16- (NK4,6,8) NK cell clusters were called significant for AS. This AS and RA separation was consistent when using different MAGMA windows (**Supplementary Figure 17**).

#### 3.2.2 UC and CD

Although fewer significant cell states were identified for UC and CD (eight and six, respectively) (**Figure 4B**), we still observed differences in pathological cell types. None of the significant cell-states were shared between UC and CD. Epithelial cells linked to UC and fibroblasts linked to CD most clearly distinguish the diseases, a finding maintained when using different MAGMA windows (**Supplementary Figure 18**). For example, we found that NK cells, CD4+ activated, and CD8+ lamina propria (LP) cells were enriched in CD compared to UC while only Tregs, CD8+ IL17+, and Cycling T cells were enriched in UC.

### 3.3 Positional SNP-gene linking methods provide greater statistical power than tested alternatives

Methods integrating scRNA-seq and GWAS summary statistics rely largely on the same preprocessing steps, yet a standardized guidance for these steps is lacking. Therefore, we evaluated the impact of inputs and preprocessing steps on results, focusing on scDRS due to its high sensitivity and covariate analysis.

**Figure 5:**
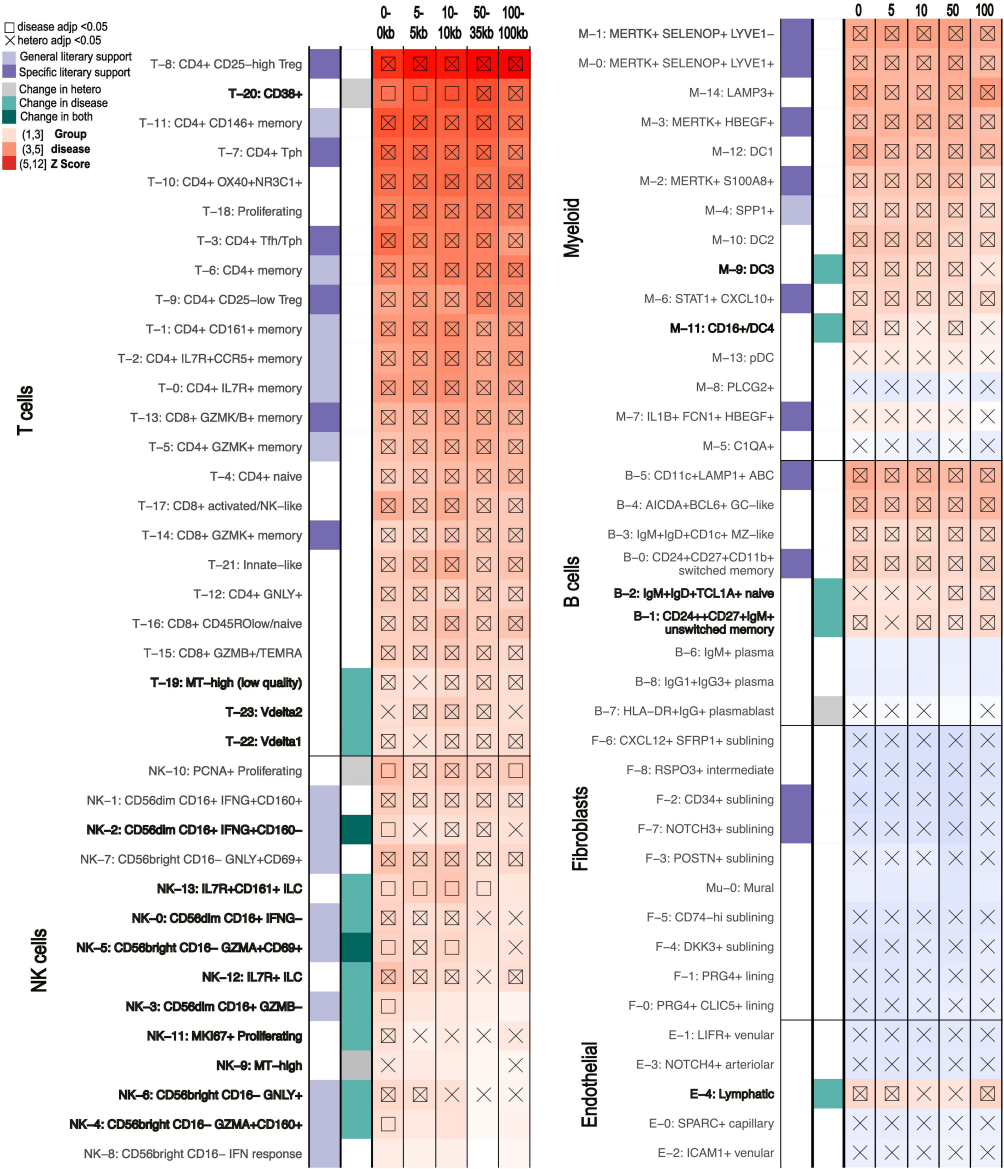
scDRS results for RA of clusters that show different levels of significance with different MAGMA windows being used to generate the GWAS inputs (0-0kb, 5-5kb, 10-10kb, 50-35kb, 100-100kb). scDRS defines significant clusters with a group disease Z-score as shown in the gradient legend (significant scores marked with square). Cell states with significant heterogeneity scores are marked by an X. General literary support means that a cell type with multiple cell states is supported by the literature while specific means a specific single cell state was supported. Cell states with changes in just scDRS disease score, heterogeneity score, or both significance calls across MAGMA windows are marked in bold and with grey or turquoise squares.

First, we considered the robustness of results when using solely positional information to connect noncoding SNPs to genes. The primary positional method to link SNPs to genes is MAGMA which relies on a window size parameter determining the distance a SNP can be from a gene to be incorporated (16). Because there is no standardization on MAGMA window size beyond the notion that a larger window size incorporates SNPs falling in cis-regulatory elements, we evaluated the impact of the most used window sizes on results (details in Methods) (4, 5, 8, 17, 49–53). Different window sizes for RA analyses only changed the significance calls for 16 of the 77 cell states in at least one of the window-sizes, half of which are only different in one window size (**Figure 5**). Importantly, none of these cell states had the top 20 group disease scores in our original results (50-35kb window). There also did not appear to be a clear pattern across the window sizes in terms of the numbers of significant cell states or the cell states changing in significance. These findings were similar with our three other diseases of study, with results for CD having the greatest differences across window sizes (**Supplementary Figures 17**, **18**). Despite only 54% of genes being shared across the top 1000 MAGMA ranked genes in all window sizes, these shared genes consistently had most of the lowest p-values (**Supplementary Figure 19**). In comparison, scGWAS showed 20 cell states with change in significance just between 10-10kb and 50-35kb window sizes in RA, including four cell states originally identified as significant by all three tools: T-22, B-5, B-0, and B-1 (**Figure 3C**, **Supplementary Figure 6**).

Given the growing concern over positional methods inaccurately assigning SNPs to genes, we next explored the usage of non-positional based data within the framework of FUMA. Although other SNP-gene linking tools can be found in **Table 3**, we focused on FUMA as a commonly used alternative to MAGMA and because it can incorporate eQTL, chromatin contact data and positional information from MAGMA to express summary statistics at the gene-level (16–20). Therefore, while FUMA uses a different summary statistics processing that doesn’t allow direct comparison to our own MAGMA based analyses, we used its MAGMA pipeline to consider the impact of alternative linkage methods (details in Methods). The 1000 genes with the lowest p-values were significantly different between positional and non-positional methods, regardless of exact summary statistics used (**Supplementary Figure 20**). When only considering genes supported from non-positional methods, 445 genes were significant, a number consistent across usual non-positional methods (**Supplementary Table 9**, **Table 3**). : The smaller number of genes was maintained regardless of p-value cutoff (**Supplementary Table 11**). Indeed, FUMA analysis that combined positional with non-positional methods showed similar results to purely using MAGMA but with only 28 of the 52 original cell states called significant (**Supplementary Figure 21**). Conversely, scDRS only lost nine and five significant cell state calls when only using the top 300 and 500 ranking genes according to MAGMA, respectively (**Supplementary Figure 21**). Only restricting scDRS to the top 100 ranking genes allowed loss of significant results at the same magnitude (23 vs 24 by FUMA) (**Supplementary Figure 22**). Still, incorporating non-positional methods added 2 significant clusters: HLA-DR+IgG+ plasmablasts (B-7) and MKI67+ Proliferating NK cells (NK-11), which were still not called significant when increasing the MAGMA window size to 100kb, a size commonly used to capture cis-regulatory element SNPs (**Figure 5**).

**Table 3:** Current methods to link SNPs to genes and the estimated number of genes output, form of significance output, and interface.

Name (Citation)	Method	Est. Gene list size	Score	Interface
cS2G (18)	Linear combination of linking scores from main S2G strategies, exon, promoter, eQTLGen, and GTEx cis-eQTL, EpiMap, ABC, and Cicero. Restricts each strategy to gene w/highest linking score.	<500 (depends on # lead variants)	cS2G score	Scripts provided
PoPs (19)	Similarity based filtering of MAGMA results (although paper described other input options).	<200 (depends on # lead variants)	PoPs score (for relative ranking)	CLI
nMAGMA (20)	Network-enhanced MAGMA links SNPs to genes by considering tissue specificity (Hi-C and eQTL) and functional interactions (WGNCA), then use MAGMA to get significance of genes.	1000+	Z-scores and P-values	Scripts provided
FUMA (17)	SNP2GENE Module: Identifies lead SNPs, can run MAGMA or map using eQTL, position, and chromatin-interaction	MAGMA based 1000+, otherwise <700	MAGMA Z-scores/P-values or min p-value of linked SNPs	Web tool
MAGMA (16)	Maps SNPs to genes via positional window, empirical gene p-value via permutation followed by PCA regression	1000+	Z-scores and P-values	CLI

All tools address linkage disequilibrium.

## 4 Discussion

In this study, we evaluated three software for linking genetics to single-cell phenotypes according to the enrichment of literature supported calls, robustness, and interpretability of results. Although all strategies identified disease-relevant cell states, single-cell based scDRS and scPagwas identified the greatest number supported by previous findings. B and T cell subsets were identified as significant for RA across all tools, aligning with the literature highlighting the disease relevance of lymphocytes (13, 14, 28, 35, 54, 55). Gene set enrichment analyses indicated the significance of monocytes and macrophages across all tools for RA, consistent with the recent work discovering the cell phenotype expanded in inflamed synovial tissue. However, only scDRS called the best defined RA induced cell states, MERTK+ myeloid cells, significant (14, 34, 35). In addition, all methods recognized autoimmune-associated B-cells (ABCs) as significant, a cell phenotype recently shown to be expanded in RA inflamed synovial tissue (14, 34, 35). Importantly, none of the algorithms identified significant fibroblast cell types despite the expansion of NOTCH3+ and CD34+ sublining fibroblasts in RA (28, 56). This finding supports previous hypotheses that these phenotypes arise only after the expansion of other genetically driven cell states called significant by scDRS (56). For UC, we found few disease-significant cell states. However, all methods identified M cells -- a recently discovered cell group with the highest expression of putative IBD risk genes in inflamed vs healthy tissue corroborated by two separate cohorts (23, 57). Interestingly, no algorithm called CD8+ IL17+ T cells despite their significantly different proportions between individuals with and without UC (23, 58). However, transcriptional changes in this group occur downstream of proportional shifts of Tregs and epithelial cells, both of which were called by scDRS (59–61).

scGWAS is more distinctly built to identify probable gene sets relevant to pathological cell states, but is significantly impacted by the pathway networks on which it bases its analyses. While removing false positives by requiring a known set of connected genes to have increased expression compared to single genes, the algorithm also assumes that the pathway file contains all possibly relevant gene connections. Therefore, true positives can be lost such as was likely with MERTK+ cells. Additionally, many of the significantly called scGWAS gene modules overlapped, depleting information content, perhaps due to the lack of cell type specificity in the pathways. This finding underscores the importance of not necessarily using the number of significant gene modules identified as a relative metric of significance for a cell type. Although scGWAS provides gene modules more conducive for certain analyses, the original network file should be considered according to a researcher’s specific focuses. In contrast, scDRS focuses on single cell based exploration by only providing genes correlated with single-cell disease scores (4). Historically, purely correlational approaches tend to be noisy and significantly impacted by data heterogeneity (62, 63). This fact might explain why both MAGMA and scGWAS genes showed relatively low correlation with single-cell disease scores, even within the annotated cell-state.

Although scPagwas uniquely integrates gene pathways with single-cell scoring, it currently has three limitations compared to scDRS. First, the computational expense of scPagwas makes scDRS far more feasible for large scale analyses; this could potentially be addressed by enabling multiprocessing for the current bottleneck in linking pathway blocks and GWAS, as done in the regression portion. Second, scPagwas currently lacks covariate adjustment, making it susceptible to batch effects, which may explain the highly polarized disease scores observed in scPagwas mitigated by scDRS. Finally, while both scDRS and scPagwas consider genes correlated with single-cell disease scores, scPagwas relies on these genes—rather than SNP-linked genes—for final cell-type analysis. Our results suggest that gene correlations can be heavily influenced by dataset heterogeneity and often poorly reflect SNP-based gene associations (e.g. MAGMA). This finding may help explain the overrepresentation of ribosomal genes among scPagwas genes despite their minimal impact on cell-state identification. Importantly, these results might also be based on the pathway size of scPagwas (default 5-300 genes); this range was optimized by the original authors but may require further optimizing for more heterogeneous datasets like those tested here. The scDRS simulated control set may also allow a more accurate prediction of significance given scDRS using scPagwas gene input, but not scPagwas, called MERTK+ cells significant despite the MERTK+ genetically enriched scPagwas pathways being linked to RA (64–67).

Importantly, the use of broad cell types, as mostly done in previous applications of scDRS, scPagwas and scGWAS, lacked the insight provided by fine-tuned cell state annotations. Indeed, all tools missed calling some cell types significant despite them calling significant cell states within them. The heterogeneity of disease scores as called significant by scDRS might indicate when a cell type, even when not called significant as a group, might contain cell states with significance. However, statistically significant heterogeneity does not always imply biological significance, as even small cell states with as few as 50 cells showed significant heterogeneity. Similarly, potential biases from including cells from diseased tissue in these atlases must be considered. For example, scDRS relies on normalized single-cell scores so statistical significance is partly driven by the comparison of cells. Despite these caveats, we were able to explain the lack of significance for certain cell states according to lack of genotypic support in the literature and their links to upstream cell states that had genotypic backing.

Given the increased sensitivity when using fine-grained cell states, we evaluated whether a single atlas could be used to assess multiple diseases. scDRS clearly distinguished between diseases with a single atlas, with literary support for the found differences from other single-cell based analyses. We were able to determine RA vs. AS and UC vs. CD pathogenesis based on the results of scDRS, using one scRNA-seq atlas for the respective comparisons. Cell states causally linked to AS according to a recent Mendelian randomization study were all called significant in AS: CD8+ activated/NK-like (T-17), pDC (M-13), and unswitched memory cells (B-1) (40). Additionally, CD8+ activated NK-like (T-17) and proliferating (T-18) T-cells showed significance here and in other studies (41, 42). NK cells were heavily implicated in AS. The unique significance of CD56dim CD16+ GZMB- cells (NK-3) in AS was supported by GZMB being expressed at much lower levels in AS patients in previous NK-focused scRNA-seq analysis and ELISA studies (43). Similarly, the significantly called IL7R+ ILC (NK-12) cell state showed similar upregulation of genes, including IL7R, as a NK cluster upregulated in AS according to previous single cell analyses (43, 44). Finally, most of the CD56bright CD16- (NK4,6,8) NK cell clusters were called significant for AS, supported by the previous findings of upregulation of CD56bright NK cells in AS (43, 44). On the other hand, epithelial cells and fibroblasts most clearly separated UC and CD respectively. Indeed, the enrichment of CD8+ LP cells, NK cells, and activated CD4+T cells has been supported by independent CD single cell analyses (45). We were also able to distinguish fibroblasts with genetic bases for CD and UC. We called RSPO3+ fibroblasts significant when multiple CD specific SNPs have previously connected this phenotype (48). Similarly, WNT2B+ fibroblasts were only called significant for CD, matching the previous finding that the group only shows genetic connection to CD despite it being expanded in both UC and CD (46, 47). Publicly available scRNA-seq data is not always available or sufficient for a certain disease, so instead researchers might need to apply the existing and relevant GWAS summary statistics to the scRNA-seq data generated from a clinically similar disease. Our findings support the ability for researchers previously constrained by the lack of appropriate scRNA-seq atlases to study diseases while not sacrificing fine-scale analyses.

Finally, we also evaluated methods incorporating noncoding SNPs for identifying pathogenic cell states. Unsurprisingly, the input gene set used can have major implications on results, regardless of the tool. We determined that MAGMA-based results in scDRS are robust to window sizes while scGWAS appeared to have larger changes. This different robustness might be explained by our finding that the genes consistent across window sizes had the highest significance scores while scGWAS considers the full list of MAGMA based scores rather than the top 1000. We also considered non-positional methods to link SNPs to genes with FUMA and found the decreased power from these tools have significant impacts on results. Non-positional methods provide significantly smaller genesets due to a focus on highly confident linkages and noisy data sources (**Table 3**). Our findings show that these low gene numbers, regardless of confidence, lead to significant decline in sensitivity. Ideally, one would be able to combine strict window MAGMA results with that of a non-positional method, however the need to combine different significance scales complicates this. The p-values output by FUMA and similar methods also often do not account for the uncertainty in the predicted SNP-gene linkages. For now, if using tools reliant on a long list of genes, we suggest focusing on cell types consistent across window sizes for MAGMA and adding genes called by other tools like FUMA. It’s important to note that regardless of the window sizes used, many SNPs were still not assigned to a gene with MAGMA. For example, with a moderately large window size of 50-35kb, about 60% of SNPs for RA and UC were linked to a gene which decreased to about 40% when that window was reduced to 10-10kb. Outside of these methods, repeating analyses with multiple GWAS summary statistics and scRNA-seq cohorts is equally relevant to ensure repeatability of results.

One way to circumvent linking SNPs to genes is using cis-regulatory elements (cREs) SNPs fall in directly. Given cRE activity is highly dependent on cellular behavior and allows accurate deconvolution of cell types, this switch could also allow separation of more nuanced cellular states (68). Additionally, tools like Cicero link cREs to their regulated genes from single cell data (69). While classic scRNA-seq data cannot capture the activity of these elements well, 5’ scRNA-seq is more sensitive to them. Moody et al. successfully applied 5’ sc-RNA-seq to detect the transcription of cREs and genes simultaneously and developed a metric to identify cell types enriched in trait heritability (10). Interestingly, they used the same summary statistics as our work for crohn’s disease (CD) and ulcerative colitis (UC). Despite using gene-based methods, we captured the same fibroblast and dendritic cell enrichment for CD that they found. However, unlike their results, we did not find an overall enrichment of T/NK cells in UC compared to CD but found some specific states in these cell types oppositely enriched and supported by the literature (45). These differences can be explained by the fact that Moody et al. relied on general lymphocyte 5’-scRNA-seq for analysis while we used scRNA-seq specifically from the colon mucosa of UC patients. The cell states we identified as seeming to conflict with findings from Moody et al. are unique to intraepithelial lymphocytes and likely would not be in their data. Overall, these results showcase the need for careful interpretation when relying on non-disease tissue specific scRNA-seq data. Exciting insight will come from evaluating the adaptation of algorithms like scDRS, scPagwas, and scGWAS to the growing cRE-based single cell data (10, 70–72).

While disease-specific and fine-scaled single-cell cRE atlases continue being developed, tools like MAGMA, scGWAS, scPagwas and scDRS provide key opportunities to identify cell states and genes associated with disease through both transcriptomics and genomics. We’ve also showed that these tools can even allow single-cell level analyses for diseases without fine-scaled sc-RNA-seq atlases currently accessible if an atlas for a similar disease is available. We note that our focus on four immunological diseases, including RA, AS, UC, and CD, may not be generalizable to all other disorders. However, these analyses represent the consistency of key genetic-relevant cell phenotypes across autoimmune disorders, providing valuable guidance for future investigations to other similar diseases. Overall, the development of tools like scDRS, scGWAS, scPagwas, along with improved SNP-Gene-cell state linking methods, are essential steps for using existing data to pinpoint the search of biological targets for treatment development.

## Data Availability

The datasets presented in this study can be found in online repositories. The names of the repository/repositories and accession number(s) can be found in the article/**Supplementary Material**.
