# Supplementary material for: Evaluating methods for integrating single-cell data and genetics to understand inflammatory disease complexity
Source: Front Immunol. 2024 Dec 5;15:1454263. doi: 10.3389/fimmu.2024.1454263 (PMC11655331; doi:10.3389/fimmu.2024.1454263)
Supplement: Supplementary file 12 [file DataSheet1.docx]

Supplementary Material

# Supplementary Notes

**Methods not considered in analysis:** CocoNet uses a similar network-based approach to the more recent scGWAS that boasts more statistical robustness. Similarly, sc-linker was published by the same laboratory as that which produced scDRS but does not perform at the single-cell level. Sc-linker instead focuses on the usage of enhancer-gene linkage methods to link SNPs to genes rather than classic positional methods; a consideration already addressed in this study.

**Gene universes:** Regardless of the tool used to map SNPs to genes, the genes must be used in both the scRNA-seq data and mapping tool. Genes identified as relevant by MAGMA or other methods may not have been considered in the scRNA-seq and therefore not be incorporated in analysis. Although only 1.2% of the MAGMA genes used for scDRS with RA had no known aliases identified in scRNA-seq genes, 18% of the top 1000 genes identified by MAGMA for UC had no known aliases found in the scRNA-seq data (Supplemental Table 5). For example, the scRNA-seq from UC did not include many genes in the 1000 top ranked by MAGMA. This list included the Mast cell growth factor *IL3*, and fibroblast growth factor *FGF21*. Both of the cell groups linked to these factors were not called significant by scDRS, possibly due to an inability to consider these genes in the analysis rather than their true lack of significance. Although remapping sequences to genomes including the annotated genes is possible, this introduces analytical differences that may disrupt the published cell state annotations and results.

**Focusing on NK-8 showing opposite results:** To assess why CD56 bright CD16- IFN response (NK-8) had been called by scGWAS while other clusters hadn’t with 50-35kb windows, we saw if the significant gene modules identified by scGWAS for this cell state show a higher expression in these cells compared to NK clusters without that same module called significant. The highest ranked cell state by scDRS, PCNA+ Proliferating (NK-10), had comparable expression of multiple NK-8 modules despite scGWAS not calling any significant gene modules for the cluster (Supplemental Figure 23). Instead, NK-8 had a higher expression of gene *STAT1* to many of the clusters, otherwise showing comparable patches of expression of the significant gene modules.

# Supplementary Figures and Tables

Supplementary Tables can be found in a single excel file labeled Supplementary_Material.xlsx where each sheet corresponds to the following:

Supplemental Table 1: Literature support for cell states in rheumatoid arthritis and ankylosing spondylitis

Supplemental Table 2: Literature support for cell states in ulcerative colitis and crohn’s disease

Supplemental Table 3: Descriptions of Packages no longer maintained or with open-source code rather than packages designed to run the full analysis

Supplemental Table 4: GWAS summary statistics sources and metadata

Supplemental Table 5: Windows used found for positional SNP-gene linking tools from literature search

Supplemental Table 6: Differences in genes available and linking for RA and UC between GWAS (based on MAGMA) and scRNA-seq before and after addressing gene aliases.

Supplemental Table 7: Significant gene modules from scGWAS, module Z score, and notes from GSEA.

Supplemental Table 8: UC GSEA C8 (cell types) top 50 gene set outputs with the top 500 genes for SCPAGWAS (with and w/o ribosomal genes), SCDRS, and MAGMA, and all significant genes for SCGWAS.

Supplemental Table 9: RA GSEA C8 (cell types) top 50 gene set outputs with the top 500 genes for SCPAGWAS (with and w/o ribosomal genes), SCDRS, and MAGMA, and all significant genes for SCGWAS.

Supplemental Table 10: The 30 genes whose expression have the highest correlation with disease scores of MERTK+ cells and the gene to which they can be paired by the original scGWAS pathway network file with the highest correlation.

Supplemental Table 11: Table of the p-value cutoffs of the genes output by either FUMA or MAGMA. The FUMA only p-values are the value minGwasP from genes.txt FUMA output while MAGMA p-values are the direct p-values from MAGMA.

## Supplementary Figures

##
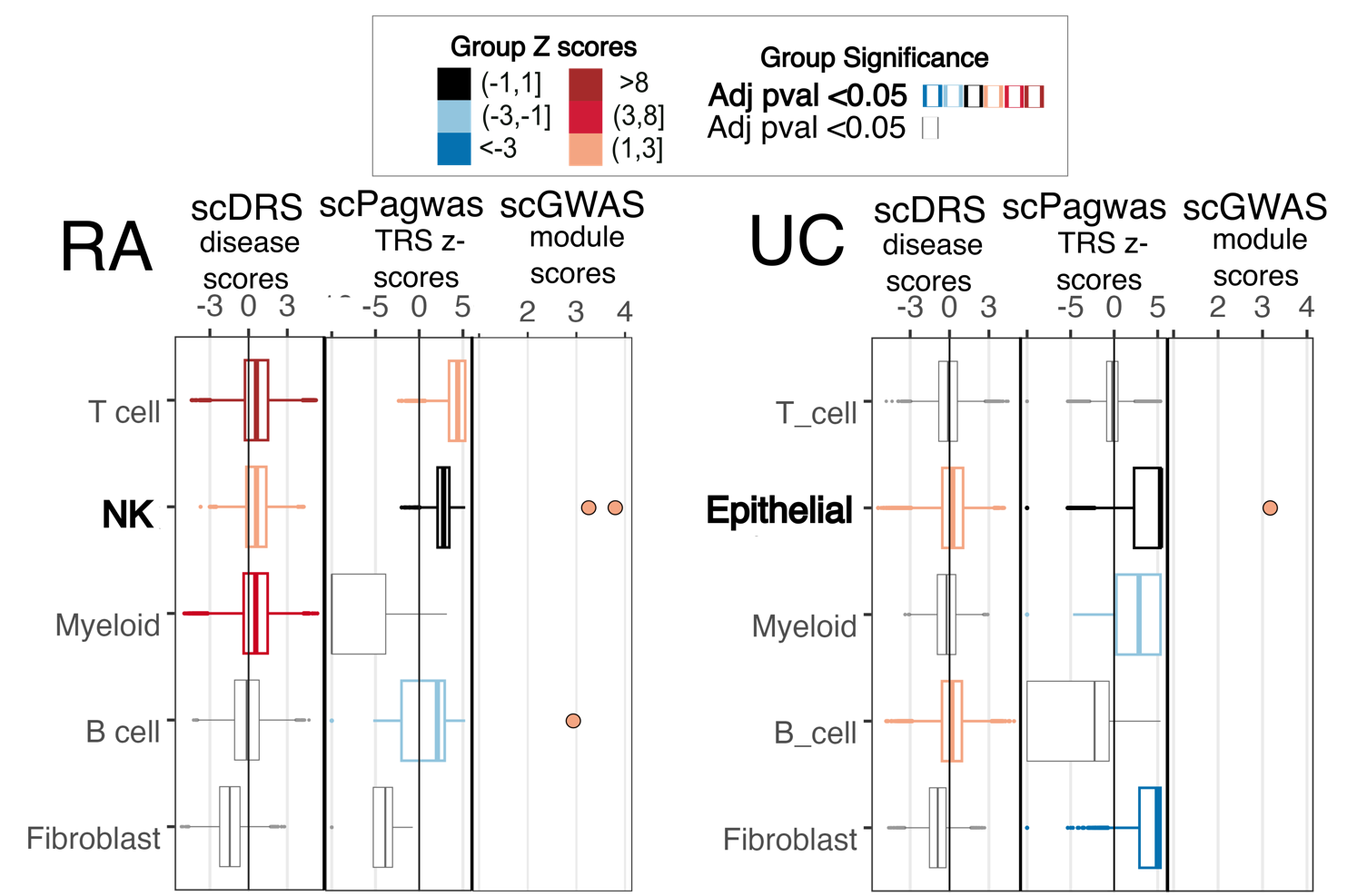


## Supplemental Figure 1. For each cell-type, the single-cell scDRS Z-scores and scPagwas TRS Z-scores are displayed in boxplots colored according to the group scDRS Z-score or group scPagwas bootstrap Z-score. Non-significant cell types are shown as non-bolded and grey, while significant cell states are bolded. scGWAS called gene modules and their disease scres are plotted with colors according to the scDRS group Z-score gradient for easier comparison. Cell types considered significant by all three tools are bolded. Left: RA (rheumatoid arthritis). Right: UC (ulcerative colitis).


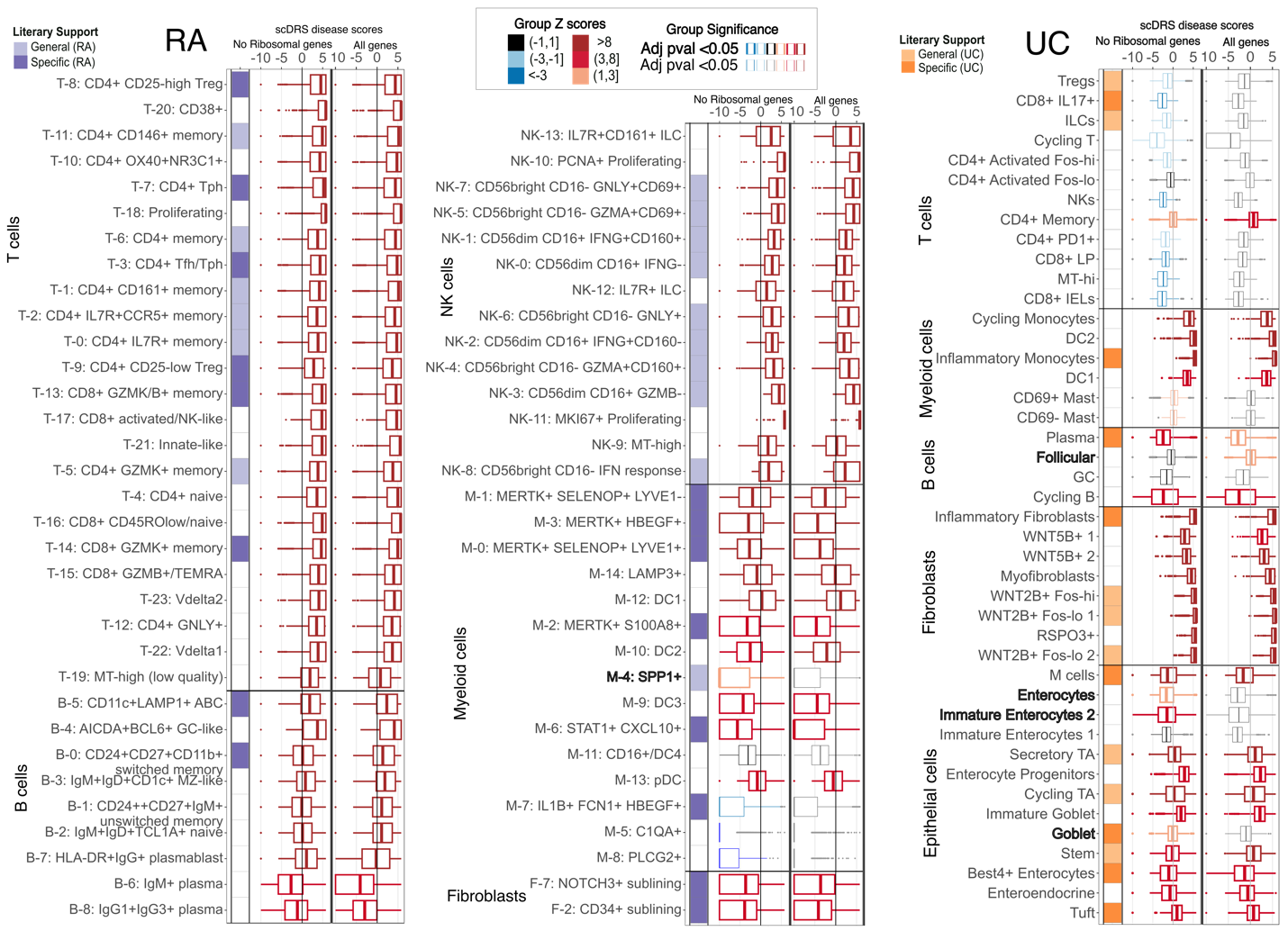


**Supplemental Figure 2.** scDRS results when using 1000 genes with the highest pearson correlation coefficients with scPagwas single-cell genetically associated disease scores, with or without ribosomal genes included. Significant calls have bolded box plots. Single cell scDRS z-scores are plotted as boxplots that are then outlined in the color of the cell group scDRS score. Cell states with different significance calls depending on ribosome gene inclusion are bolded.


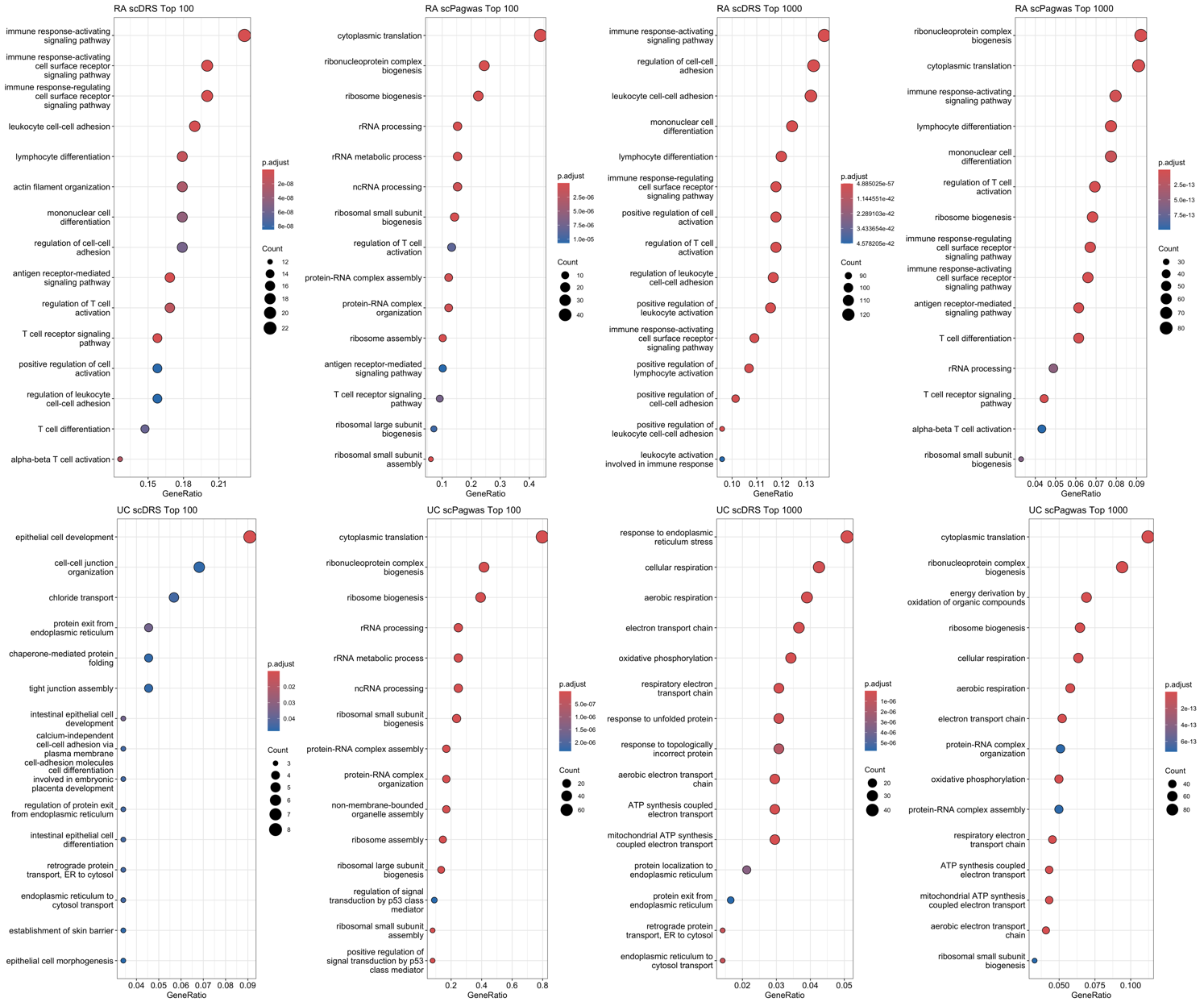


**Supplemental Figure 3.** Top 15 Gene Ontology results for the top 100 and 1000 ranked genes according to correlation with scDRS disease scores and scPagwas genetically associated pathway activity scores. Top is RA and Bottom is UC.


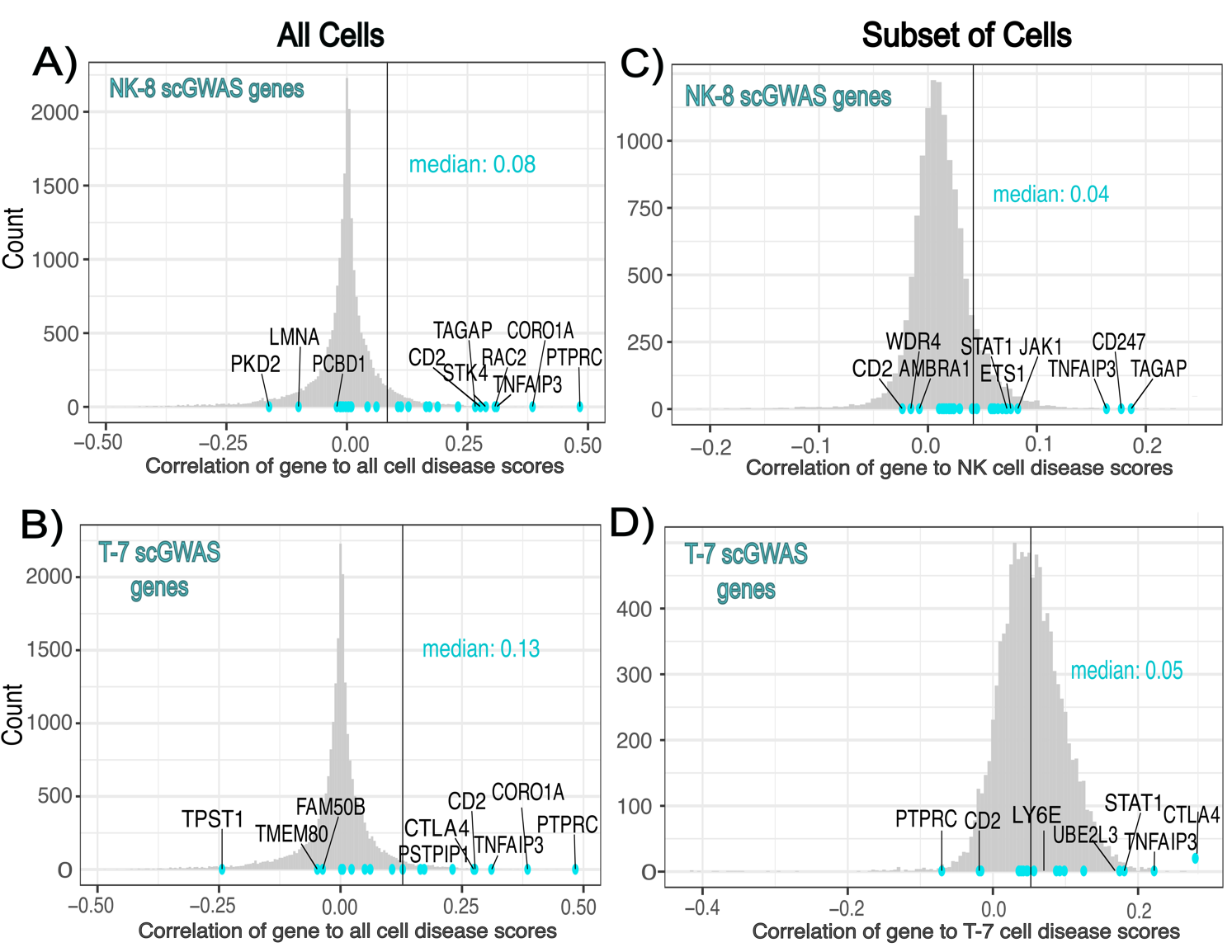


**Supplemental Figure 4.** Histograms of the correlations of all studied genes with scDRS disease scores and scPagwas gPA scores (grey) in all cells (**A,B),** NK cells **(C),** or T-7 cells **(D)** with the appropriately labeled scGWAS module genes highlighted (turquoise). Median correlation score of scGWAS genes is written and shown as a vertical line. scGWAS genes with highest and lowest scores are labeled.

**
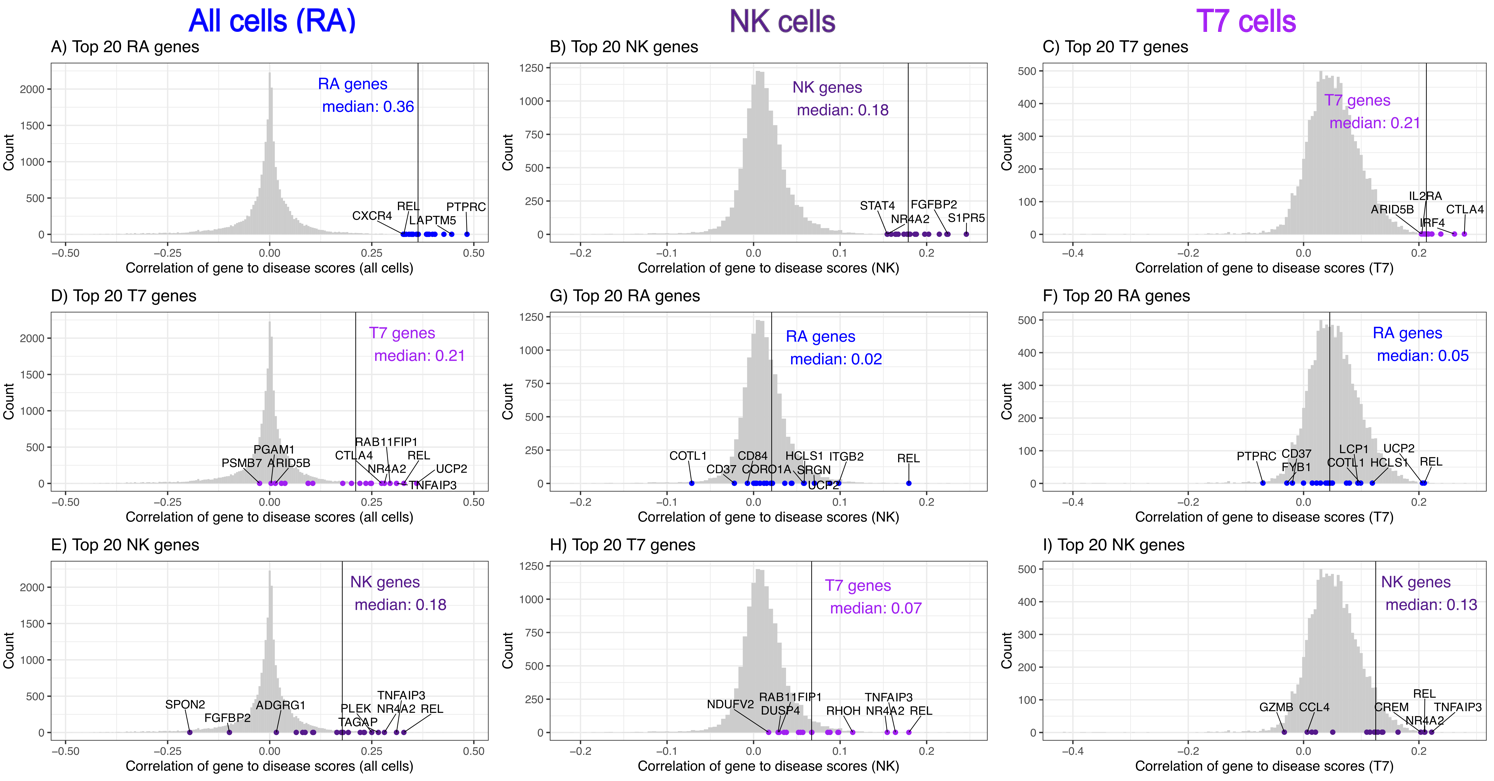
Supplementary Figure 5.** Histograms of the scDRS correlation scores of the top 20 genes correlated with disease scores in all cells (RA), NK cells, or T-7 cells, within the different cell type options. The genes with the highest and lowest scDRS correlations within the top 20 list are annotated. The median correlation of the top 20 genes are listed. All data is from the RA analysis.


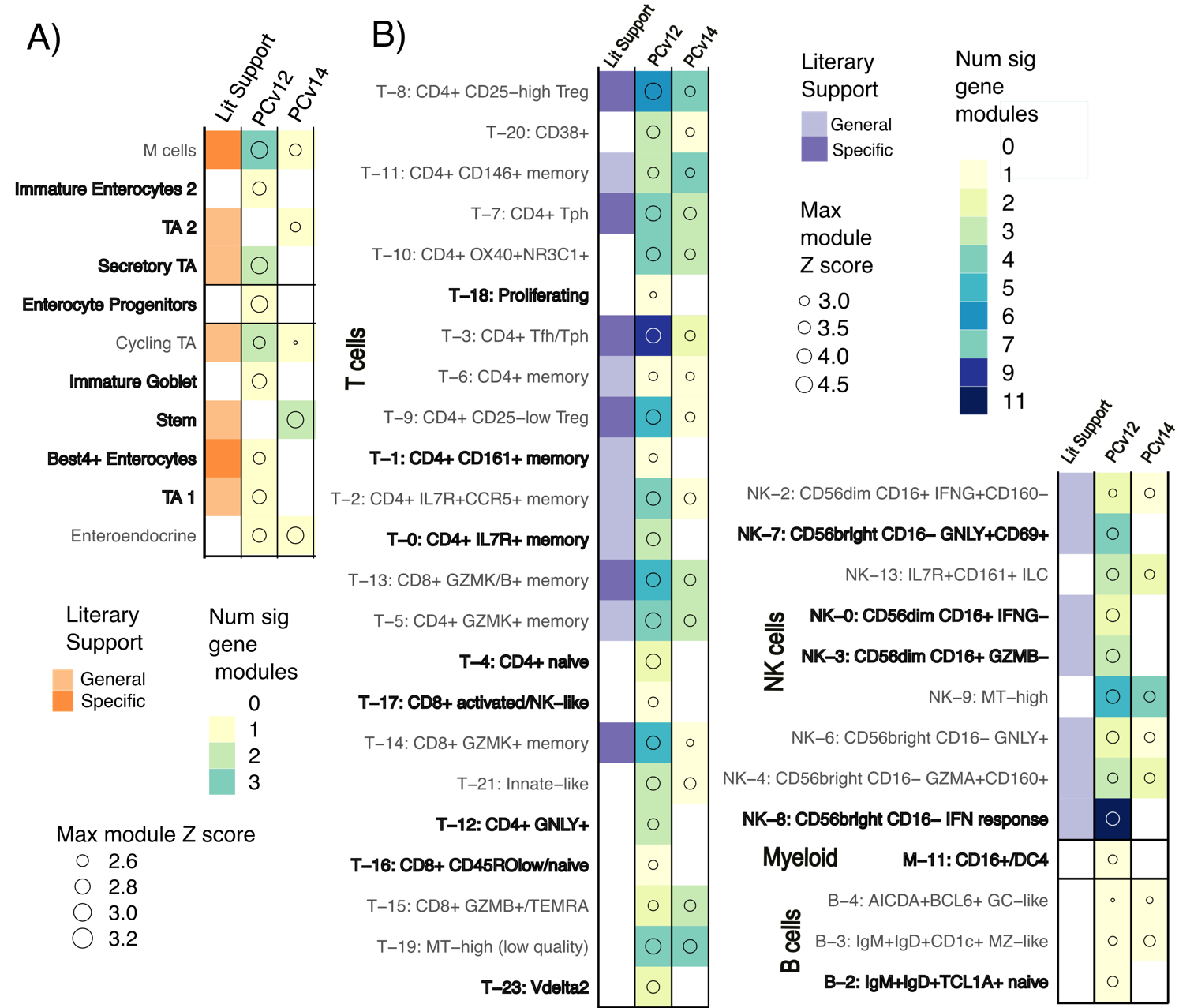


**Supplemental Figure 6.** scGWAS results when using a pathway file based on Pathway Commons v12 or 14 for Ulcerative Colitis with 10kb-10kb MAGMA windows (A) and Rheumatoid Arthritis with 50-35kb MAGMA windows (B). Results are highlighted according to the number of significant gene modules called per RA cell state and max disease Z score across the modules for each cell state. Only cell states with a significant gene module from using either pathway file are shown. Cell states without a significant gene module called when only one of the pathway files was used are bolded. Max module Z score refers to the maximum z score value of all significant gene modules called for a cell state.


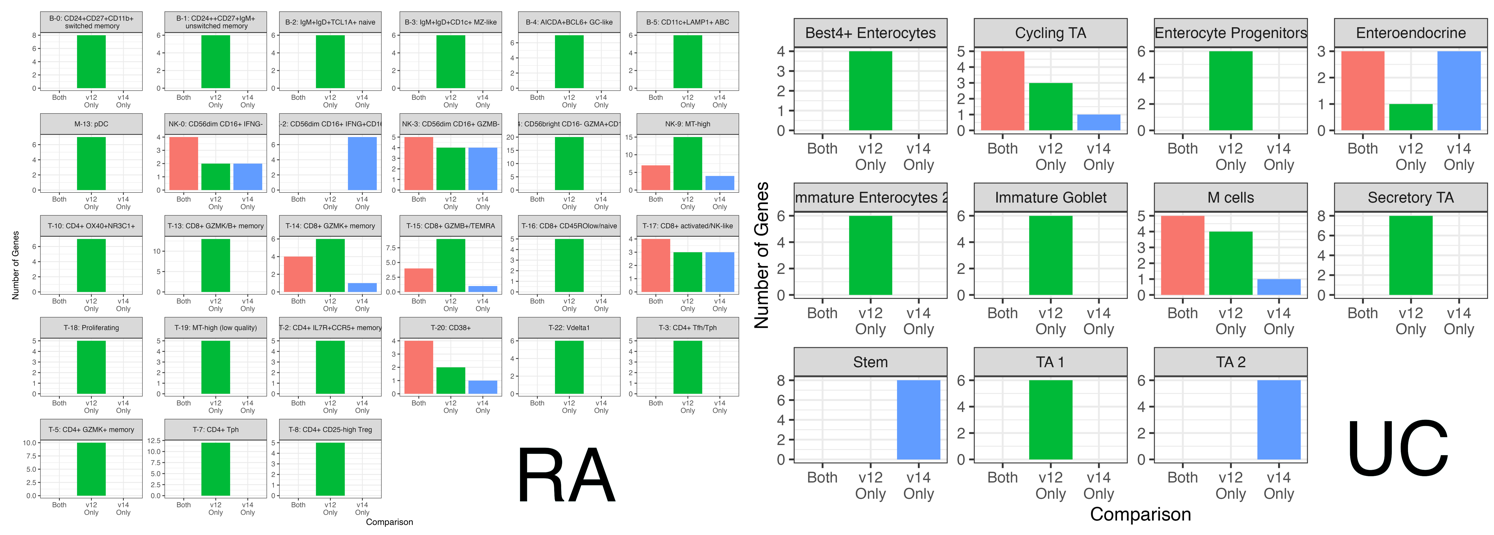


**Supplemental Figure 7.** The number of genes of significant modules of RA and UC cell states called when using input from Pathway Commons v12 (v12 Only), v14 (v14 Only), or either (Both) for 10-10kb MAGMA windows. Only cell states with significant gene modules are shown.


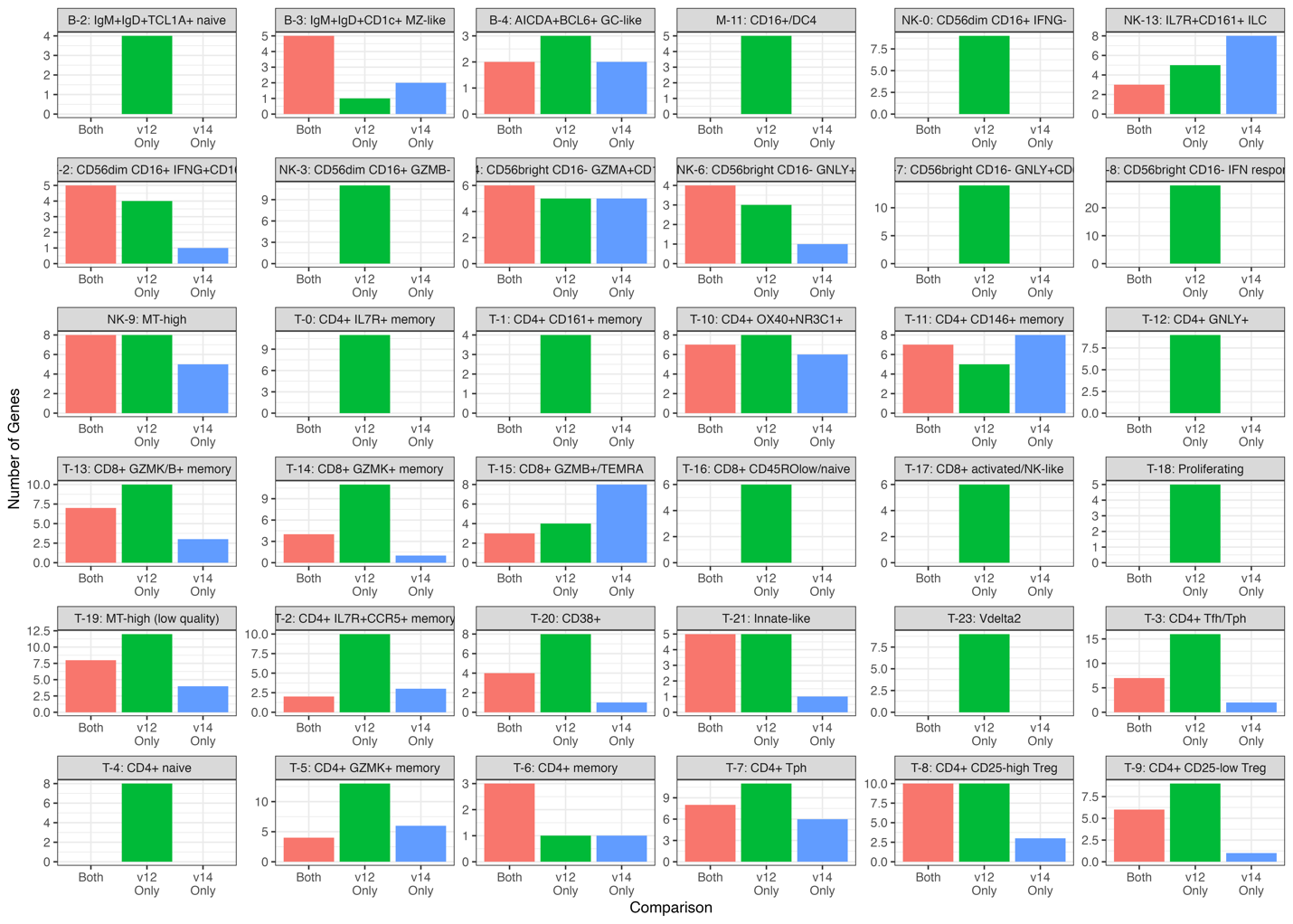


**Supplemental Figure 8.** The number of genes of significant modules of RA cell states called when using input from Pathway Commons v12 (v12 Only), v14 (v14 Only), or either (Both) for 50-35kb MAGMA windows. Only RA cell states with significant gene modules are shown.


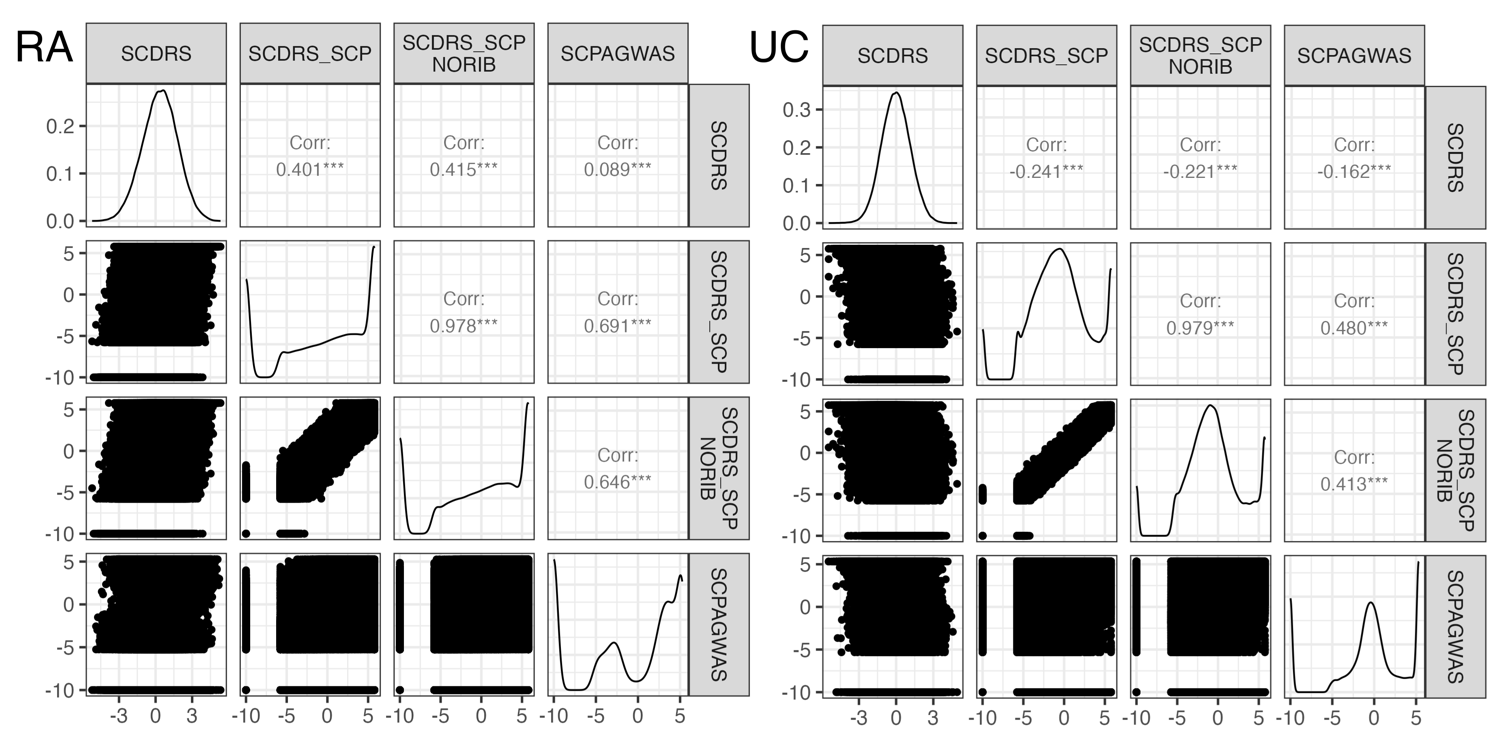


**Supplemental Figure 9**. Distribution and correlation of scDRS z-scores with MAGMA input (SCDRS), scPagwas gene input (SCDRS_SCP), scPagwas gene input without ribosomal genes (SCDRS_SCP NORIB), and scPagwas z-scores for trait relevant scores (SCPAGWAS). Diagonals show the distributions of the scores and correlation coefficients were calculated with the Spearman method using base R function cor. Left: Rheumatoid arthritis (RA), Right: Ulcerative colitis (UC).


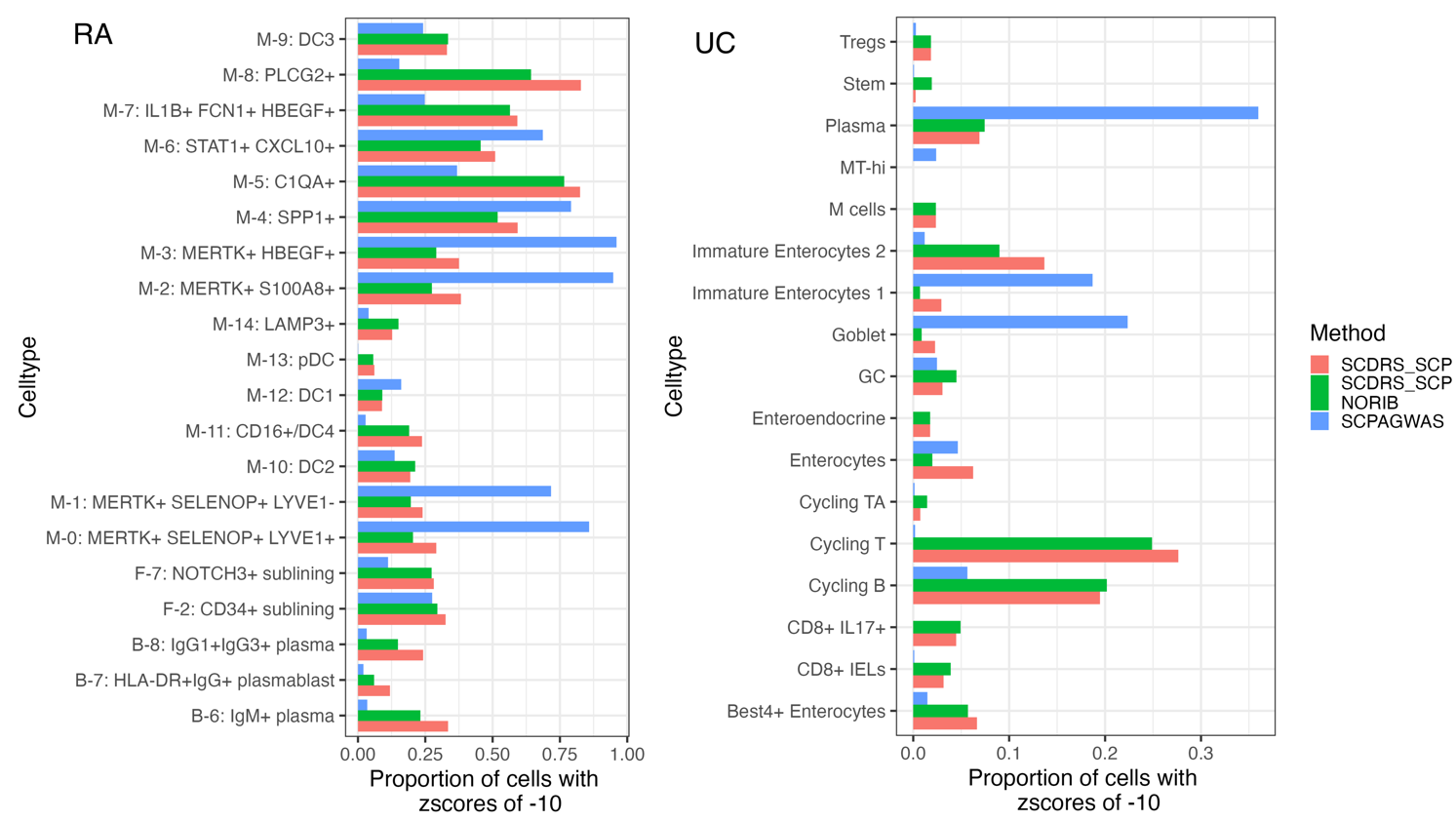


**Supplemental Figure 10.** The proportion of cells within each cell type that have disease zscores of -10 according to scDRS z-scores using scPagwas gene input (SCDRS_SCP) or scPagwas gene input without ribosomal genes (SCDRS_SCP NORIB), and scPagwas z-scores for trait relevant scores (SCPAGWAS). Left: Rheumatoid arthritis (RA), Right: Ulcerative colitis (UC).


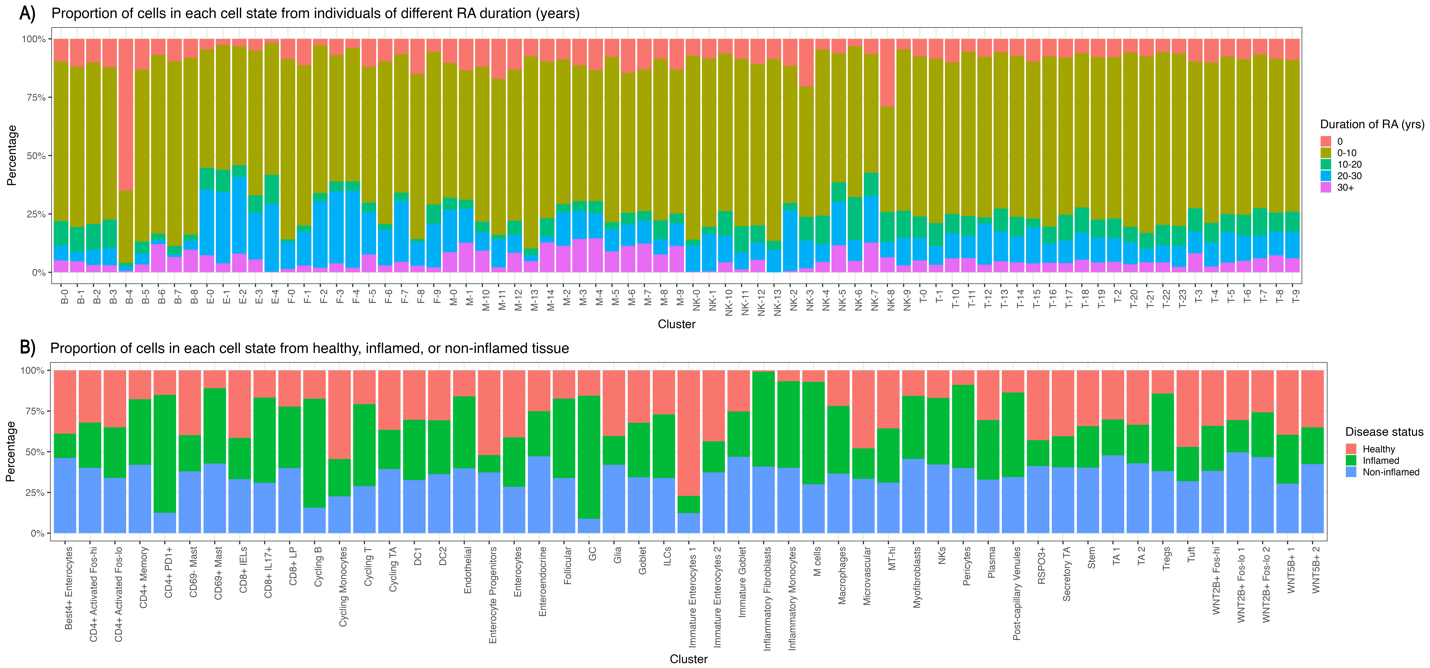


**Supplementary Figure 11.** **Proportion of disease status across cell-states.** The proportion of cells belong in either: A) RA duration for RA and B) disease tissue status for UC were graphed for each of the relevant cell states.


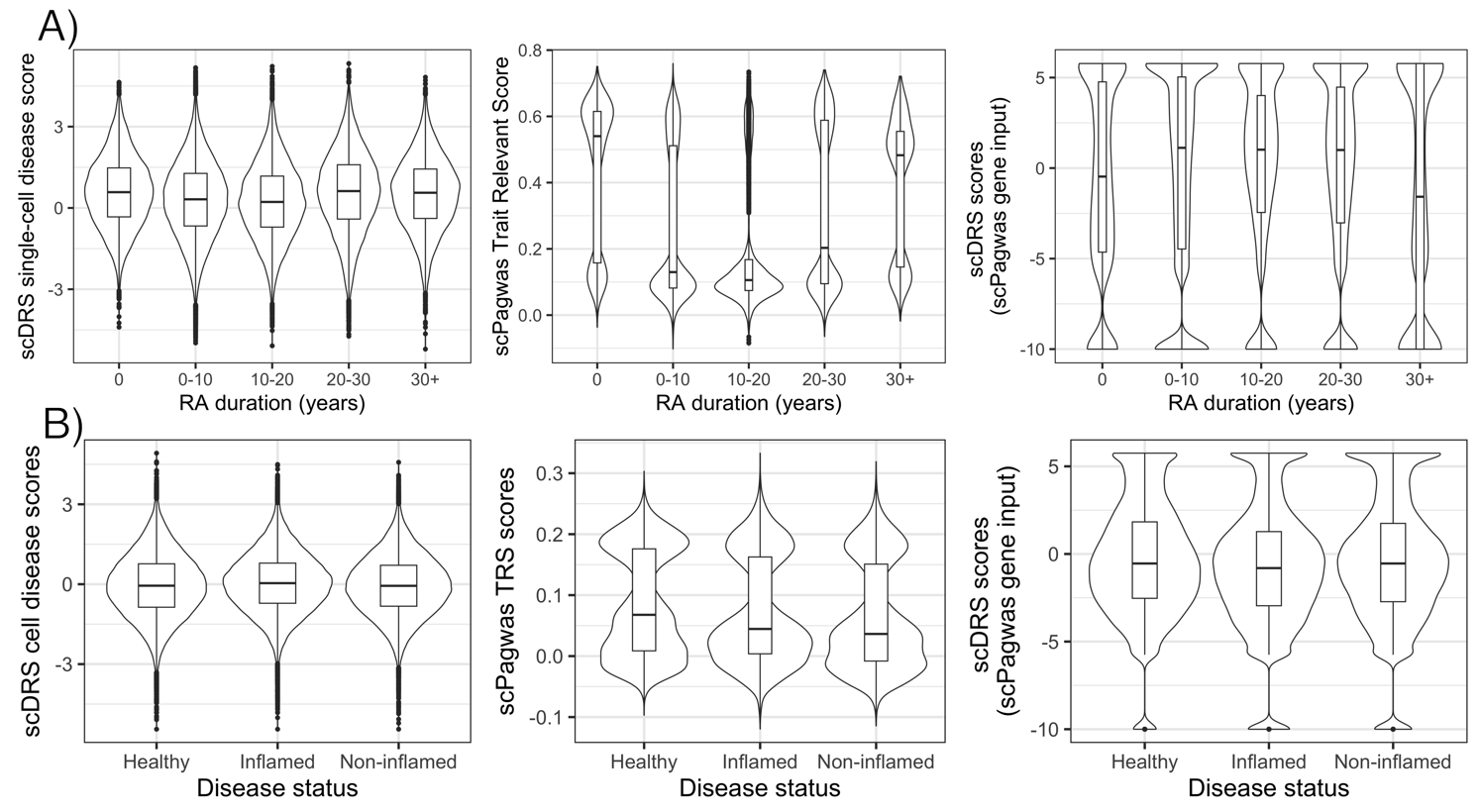


**Supplemental Figure 12. Single-cell disease scores connection to disease status.** Single- cell disease scores (from scDRS, scDRS with scPagwas input, and scPagwas) were graphed for cells belonging in difference disease annotations: A) RA duration for RA and B) disease tissue status for UC. ANOVA was followed by Tukey multiple comparison analysis (95% confident intervals).


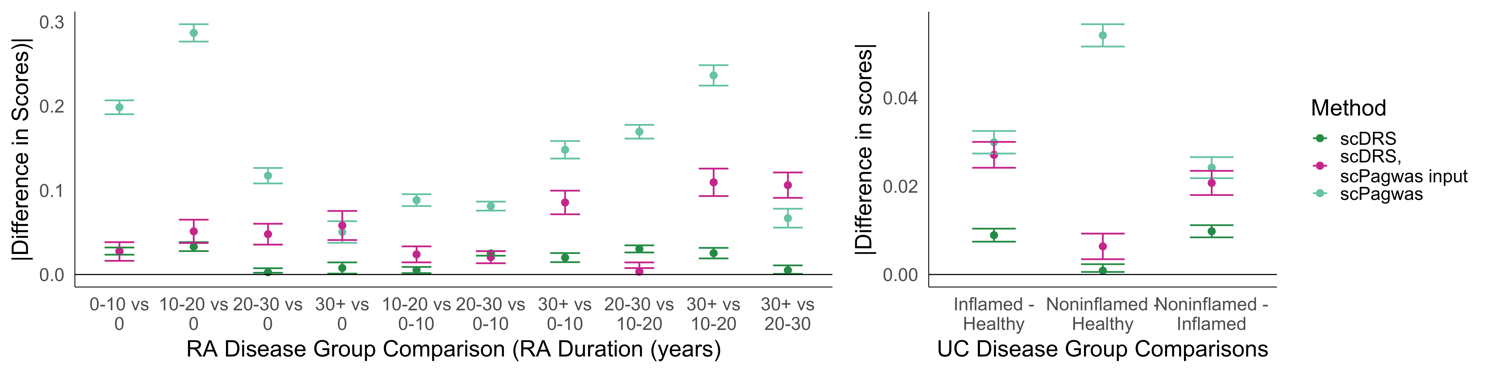


**Supplemental Figure 13. scDRS improves covariate bias**. Confidence intervals around mean differences (absolute value) from Tukey-based ANOVA post-hoc comparison analysis of cell disease scores (from scDRS, scDRS with scPagwas input, and scPagwas) according to the disease status of cells (RA duration in years for RA (left) and Inflammation status for UC (right)). Confidence intervals touching the 0.0 line indicate nonsignificant differences in the cell disease scores between the compared groups. Single cell disease scores were scaled from 0 to 1 using min-max scaling to allow easier comparison of differences. Exact Tukey and ANOVA results can be found at our github under (SCRNA-GWAS-Benchmarking/analysis/0A_scGWAS_scDRS/Sensitivity).

**
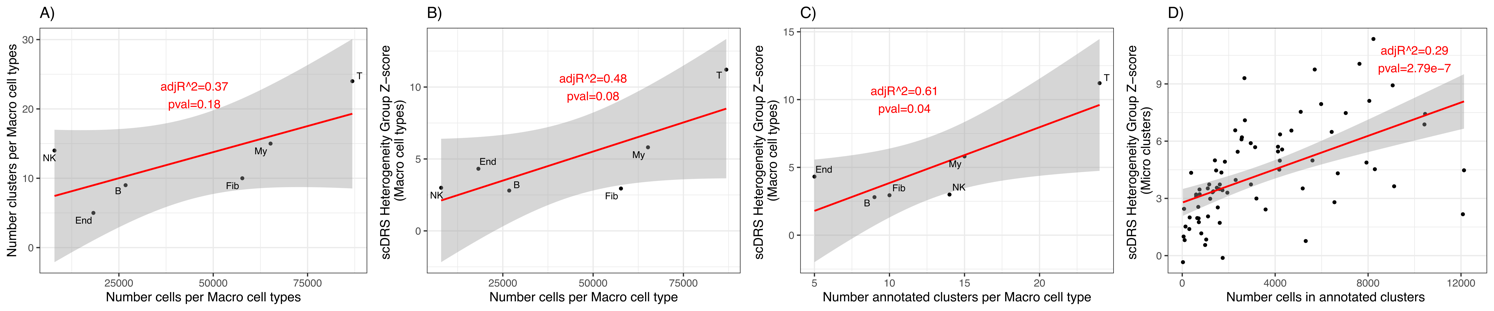
Supplemental Figure 14.** Linear regression between heterogeneity score and number of clusters/cells in RA scRNA-seq data. The adjusted R^2^ and model p-value (F-test) are included. Left: the number of cells in Macro-cell types (T-cell, B-cell, Myeloid (My), NK, Fibroblast (Fib), Endothelial (End))  and their number of annotated clusters (A) and group scDRS disease score heterogeneity z-scores (B) (N=6). C) Number of annotated clusters in large-cell types and group scDRS disease score heterogeneity z-scores (N=6). B) the number of clusters in large-cell types and their group scDRS disease score heterogeneity z-scores. D) Number of cells in annotated clusters, and their group heterogeneity z-scores (N=77). Details of the linear regression results can be found in the jupyter notebook heterogeneity.ipynb on github. MAGMA window of 50-35kb was used.

**
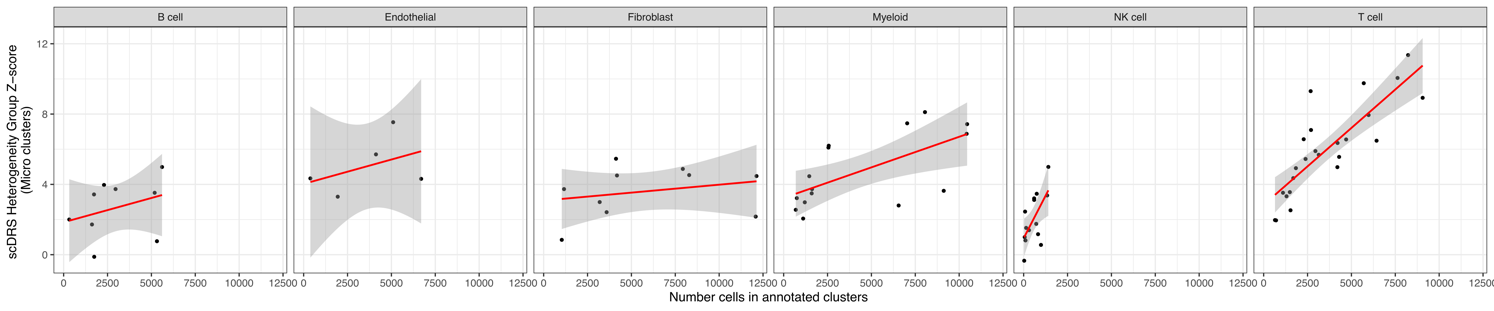
Supplemental Figure 15.** Linear regression between heterogeneity score and number of cells in each cluster annotated from RA scRNA-seq data, separated by cell type. The adjusted R^2^ and model p-value (F-test) are included. Details of the linear regression results can be found in the jupyter notebook heterogeneity.ipynb on github. MAGMA window of 50-35kb was used.

**
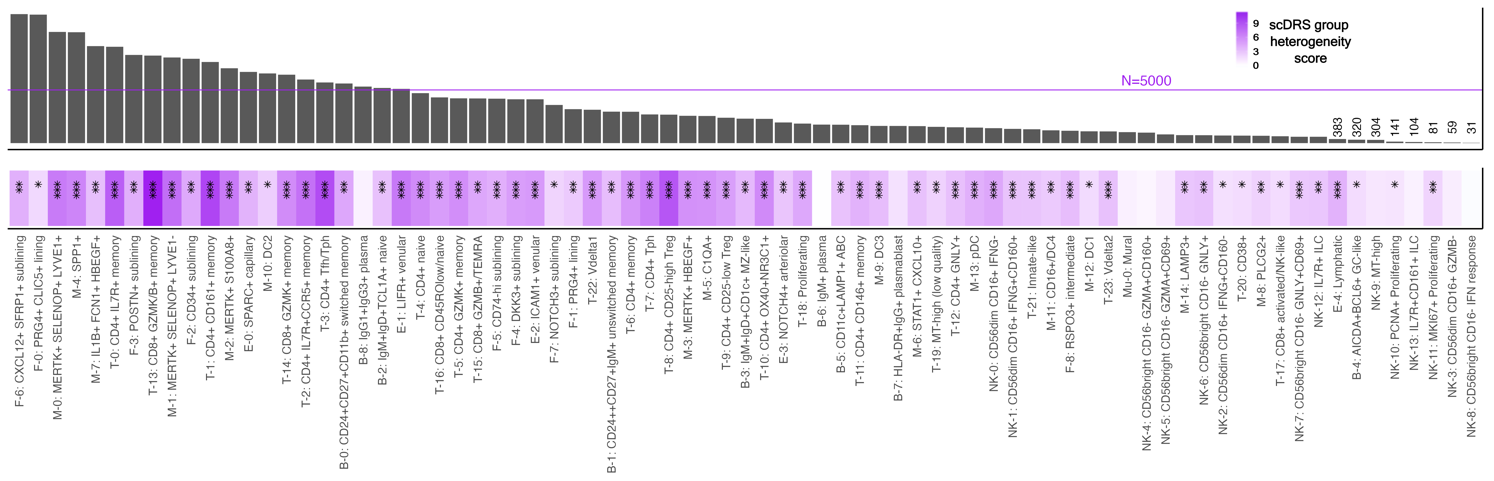
Supplemental figure 16:** The scDRS group heterogeneity scores [of disease scores] of cell clusters from RA and the size of said clusters. Any cluster below 500 cells is noted by the number of cells while the rest have a N=5000 bar for reference with the largest number being 12k. Significance legend: *P<0.05, **P<0.01, ***P<0.001. MAGMA window of 50-35kb was used.

**
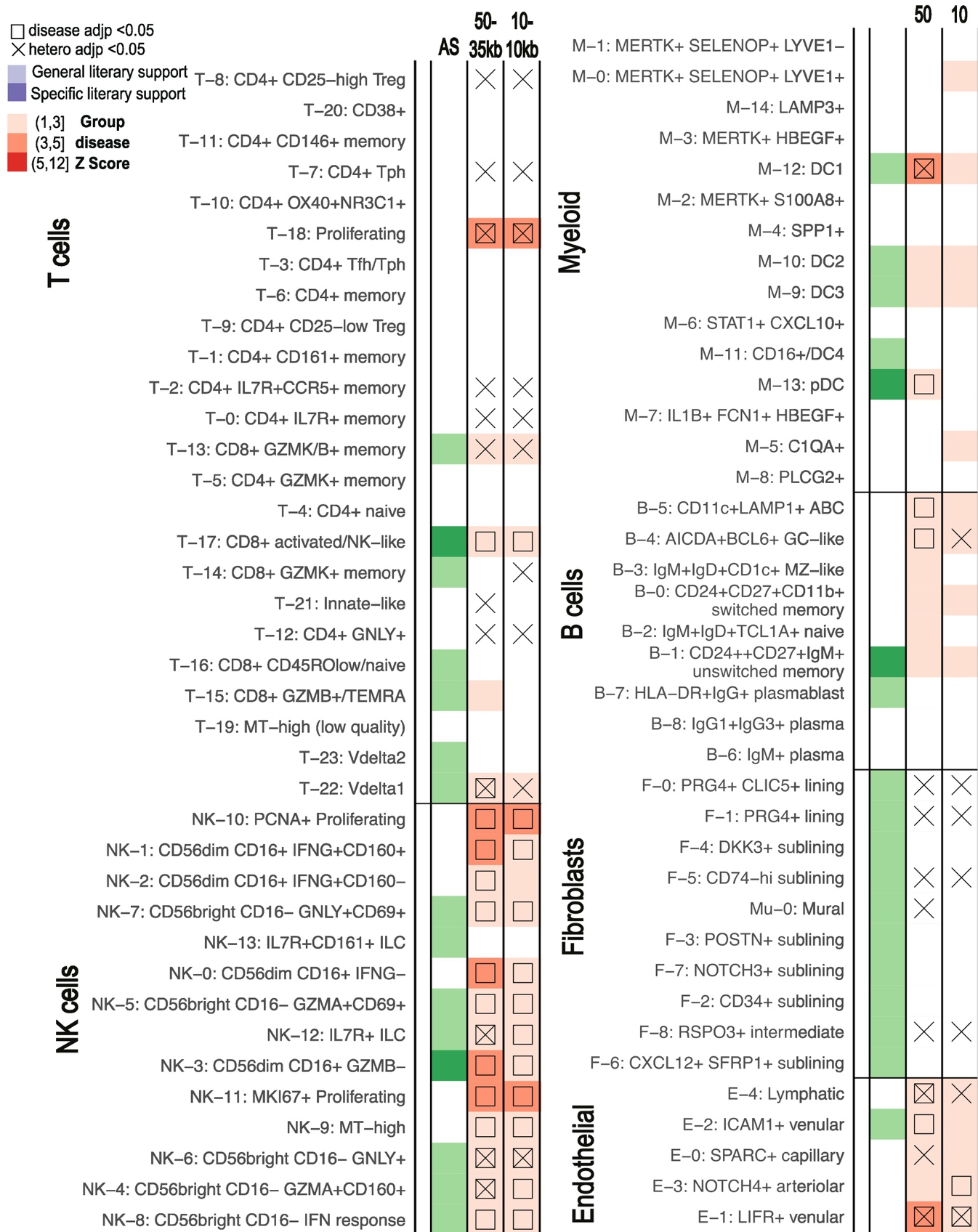
**

**Supplemental Figure 17. MAGMA window comparisons for ankylosing spondylitis.** scDRS results of significant clusters for AS using 50-35kb and 10-10kb windows. scDRS defines significant clusters with a group disease Z-score as shown in the gradient legend. AS = ankylosing spondylitis.

**
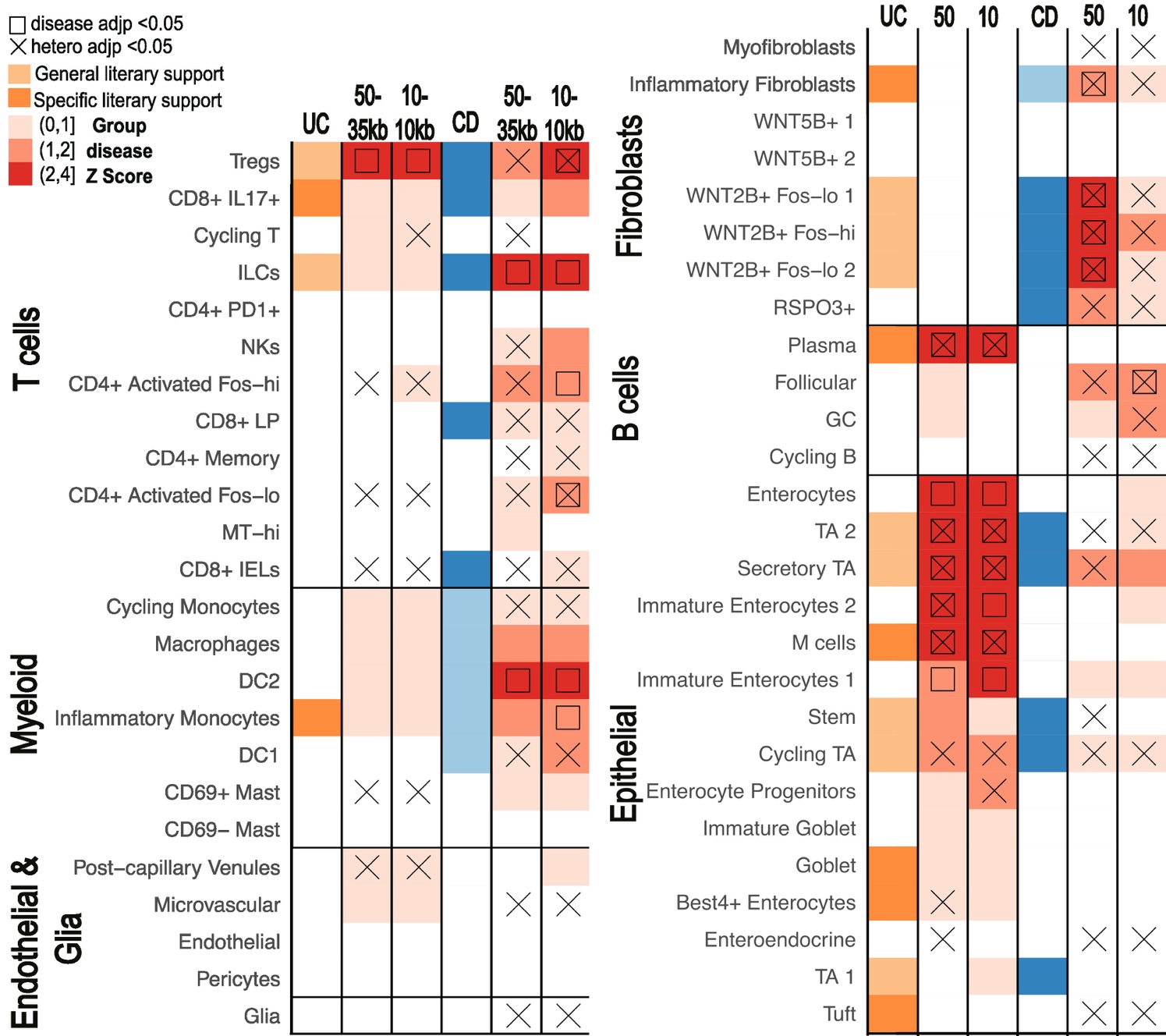
Supplemental Figure 18. MAGMA window comparisons for ulcerative colitis and crohn's disease.** scDRS results of significant clusters for UC and CD using 50-35kb and 10-10kb windows. scDRS defines significant clusters with a group disease Z-score as shown in the gradient legend. Significance legend: *P<0.05, **P<0.01, ***P<0.001. UC=ulcerative colitis, CD=crohn’s disease.

**
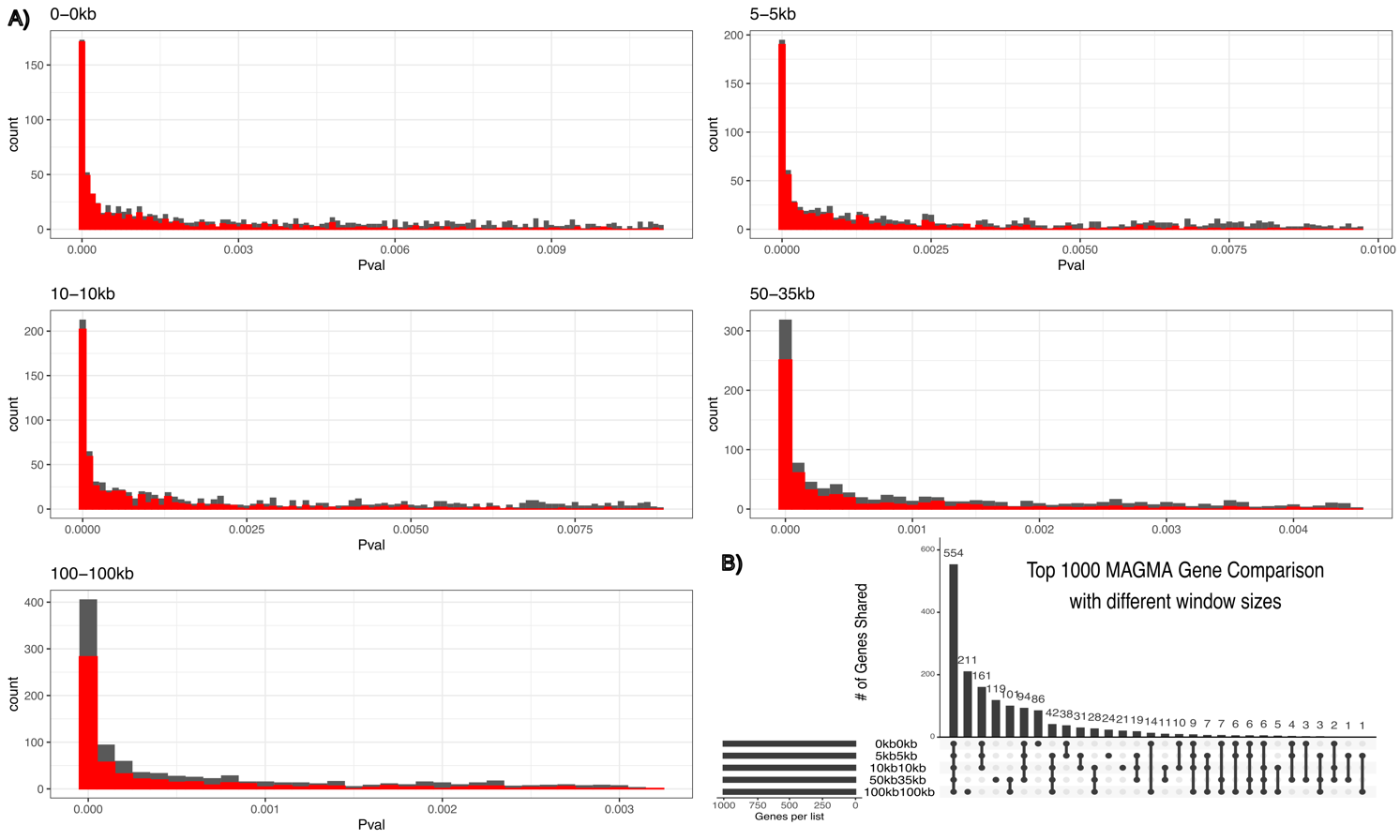
Supplementary Figure 19.** The number of genes shared between the top 1000 genes deemed significant by MAGMA that also were found in scRNA-seq, with 5 different windows (0kb-0kb, 5kb-5kb, 10kb-10kb, 50kb-35kb, 100kb-100kb) **B)** The distribution of p-values of the the same top 1000 genes in B) with the distribution of the shared 554 genes highlighted in red.

**
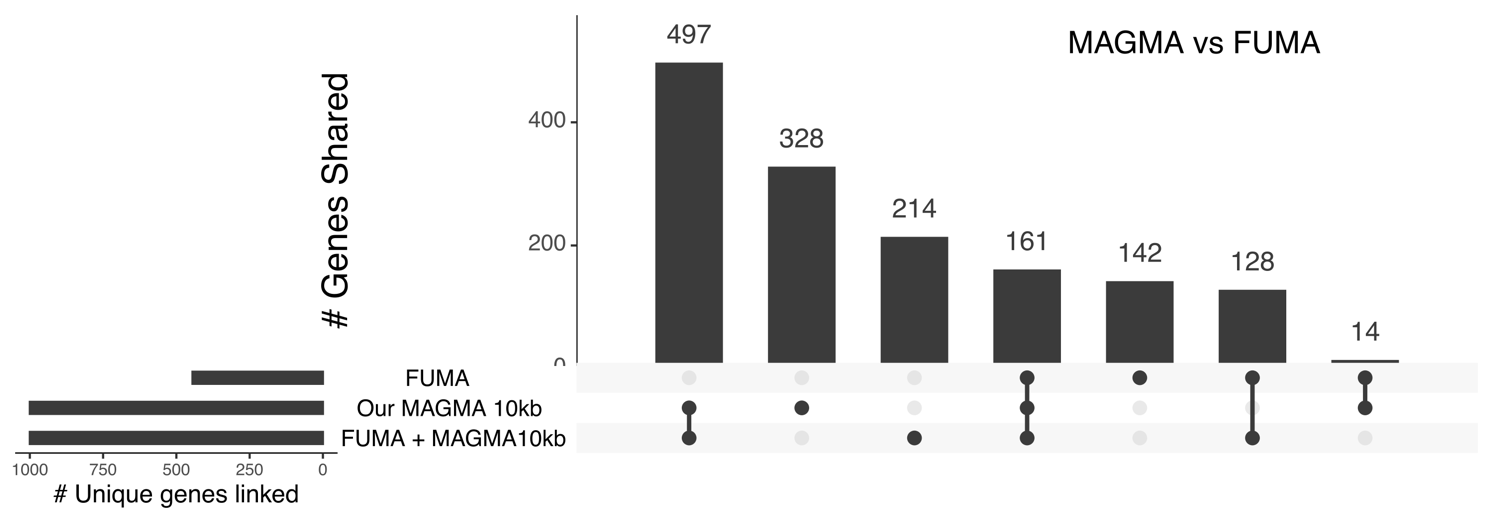
Supplementary Figure 20:** UpSet plot of the mapped genes according to FUMA (FUMA), MAGMA run on FUMA’s final summary statistics (FUMA+MAGMA10kb), and MAGMA run on our final summary statistics (Our MAGMA 10kb).

**
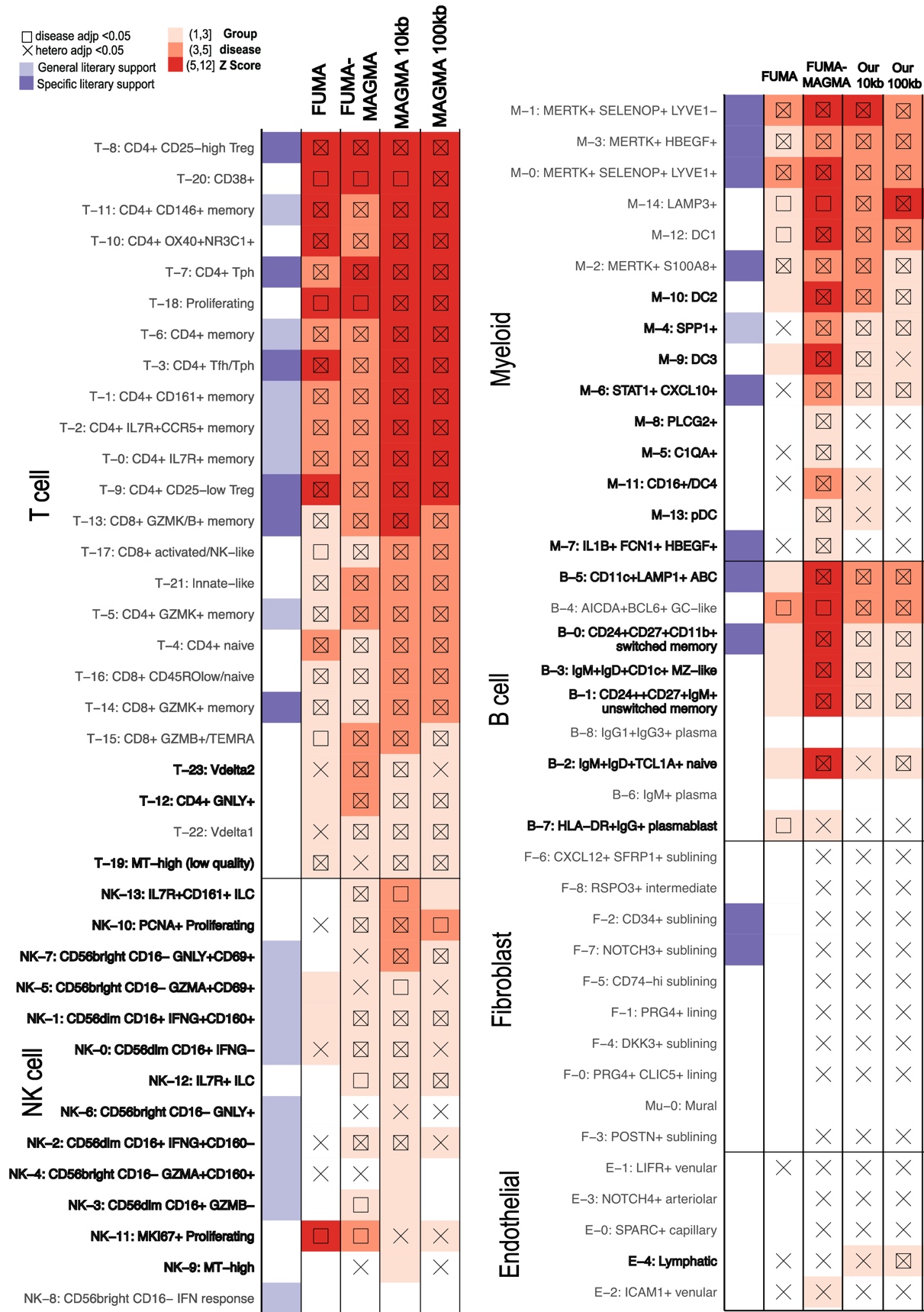
**

**Supplementary Figure 21:** scDRS results of significant clusters from rheumatoid arthritis calls using inputs from FUMA generated SNP analysis using MAGMA 10kb window mapping (FUMA-MAGMA), using FUMA based mapping including 10kb window, eQTL, and 3D chromatin interaction mapping (FUMA), and the 10kb/100kb MAGMA windows from Figure 5. Cell states with literary support are highlighted in purple. Cell states with differences in disease significance calls are bolded.


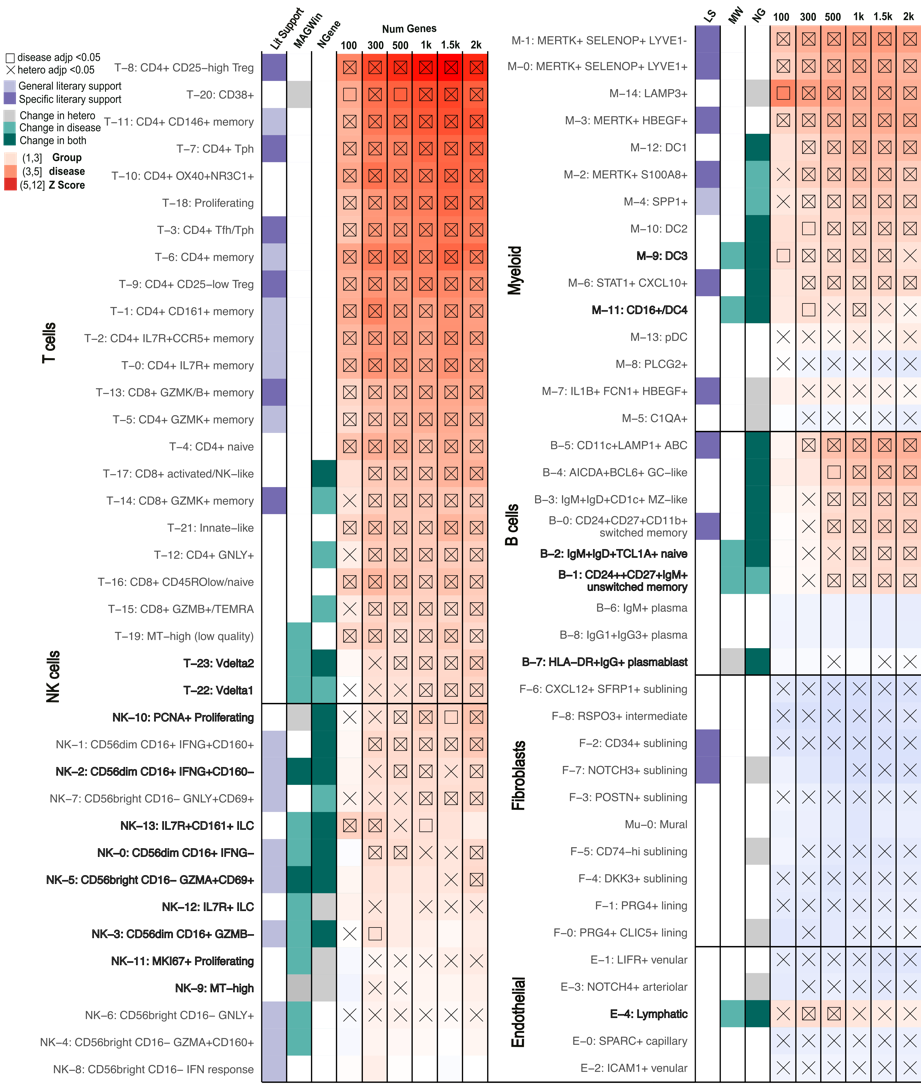


**Supplemental Figure 22**. scDRS results for RA of cell-states that show different levels of significance with a different number of top ranked MAGMA genes (100, 300, 500, 1000, 1500, and 2000). Cell states with significant disease scores and heterogeneity scores are marked by a box or an X, respectively. General literary support means that a cell type with multiple cell states is supported by the literature while specific means a specific single cell state was supported*.* Cell states with changes in just scDRS disease score, heterogeneity score, or both significance calls across MAGMA windows (MAGWin) or number of top-ranked genes (NGene) are marked with grey or turquoise squares. Cell states with changes both across MAGMA windows and number of top-ranked genes are bolded.

**
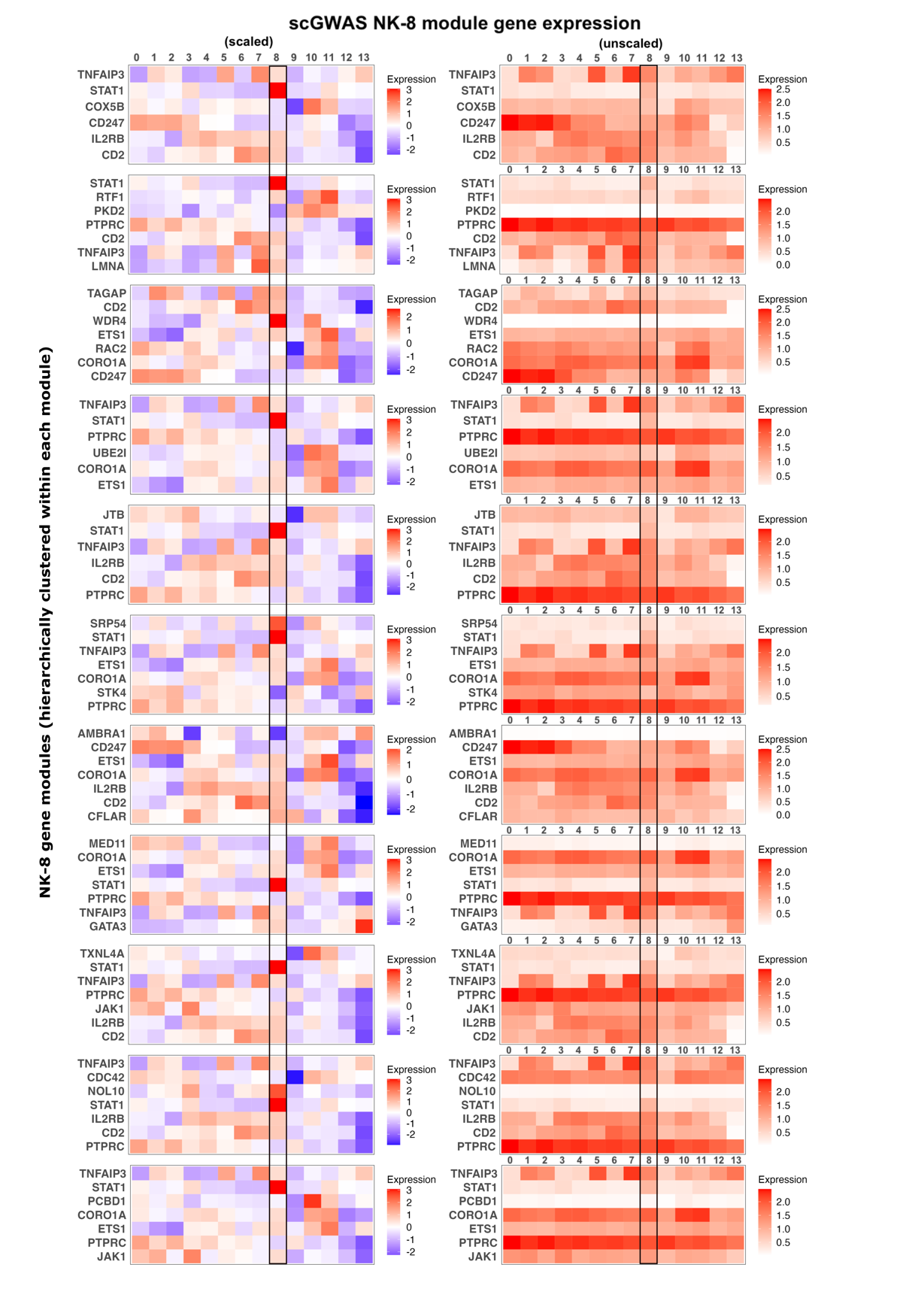
**

**Supplemental Figure 23. Heatmaps of expression of NK scGWAS significant modules genes.** Mean expression of the 11 NK-8 significant module genes according to scGWAS in both scaled expression and raw mean expression (counts) for the 14 NK clusters.
